# The Extracellular Matrix Environment of Clear Cell Renal Cell Carcinoma Determines Cancer Associated Fibroblast Growth

**DOI:** 10.3390/cancers13235873

**Published:** 2021-11-23

**Authors:** Kyle H. Bond, Takuto Chiba, Kieran P. H. Wynne, Calvin P. H. Vary, Sunder Sims-Lucas, Jeannine M. Coburn, Leif Oxburgh

**Affiliations:** 1 Rogosin Institute, 310 East 67th St., Room 2-43, New York, NY 10065, USA; kyb9010@nyp.org; 2 Graduate School of Biomedical Science and Engineering, University of Maine, 5775 Stodder Hall, Room 44S, Orono, ME 04469, USA; 3 Children’s Hospital of Pittsburgh, 4401 Penn Ave., Rangos Research Building, Pittsburgh, PA 15224, USA; chibat@pitt.edu (T.C.); Sunder.Sims-Lucas@chp.edu (S.S.-L.); 4 Maine Medical Center Research Institute, 81 Research Drive, Scarborough, ME 04074, USA; kieran.wynne1@ucd.ie (K.P.H.W.); VaryC@mmc.org (C.P.H.V.); 5 Systems Biology Ireland, University College Dublin, Belfield, Dublin 4, Ireland; 6 BME Department, Worcester Polytechnic Institute, 60 Prescott St., Worcester, MA 01605, USA; jmcoburn@wpi.edu

**Keywords:** collagen VI, collagen XII, periostin, fibronectin, tenascin C, HSPG2, TGFBI, lumican, laminin

## Abstract

**Simple Summary:**

Clear cell renal cell carcinoma (ccRCC) is the most common kidney cancer. Cell culture studies have the potential to explain how interactions between cancer cells and their support cells (stroma) determine growth and malignancy of ccRCC. The ability to grow tumor cells together with stroma from patient tumors is critical for studies of these interactions, but conventional culture methods do not provide representation of both cell types. We hypothesized that mimicking the extracellular environment of the tumor would promote growth of both tumor and stromal cells. We characterized the extracellular protein composition of patient ccRCCs and defined a nine-component protein blend to mimic the tumor microenvironment. Adherence of tumor cells, supporting stromal cells, and immune cells was demonstrated. Furthermore, we found that cells from patient tumors grown in our protein blend maintained representation of both tumor cells and cancer-associated fibroblasts (CAFs), a stromal cell type that plays a defining but poorly understood role in establishing the tumor microenvironment. This study demonstrates the dependence of CAFs on the extracellular protein composition and provides a technology to investigate interactions between tumor cells and CAFs isolated from patient ccRCCs.

**Abstract:**

Clear cell renal cell carcinoma (ccRCC) is the most common kidney cancer and is often caused by mutations in the oxygen-sensing machinery of kidney epithelial cells. Due to its pseudo-hypoxic state, ccRCC recruits extensive vasculature and other stromal components. Conventional cell culture methods provide poor representation of stromal cell types in primary cultures of ccRCC, and we hypothesized that mimicking the extracellular environment of the tumor would promote growth of both tumor and stromal cells. We employed proteomics to identify the components of ccRCC extracellular matrix (ECM) and found that in contrast to healthy kidney cortex, laminin, collagen IV, and entactin/nidogen are minor contributors. Instead, the ccRCC ECM is composed largely of collagen VI, fibronectin, and tenascin C. Analysis of single cell expression data indicates that cancer-associated fibroblasts are a major source of tumor ECM production. Tumor cells as well as stromal cells bind efficiently to a nine-component ECM blend characteristic of ccRCC. Primary patient-derived tumor cells bind the nine-component blend efficiently, allowing to us to establish mixed primary cultures of tumor cells and stromal cells. These miniature patient-specific replicas are conducive to microscopy and can be used to analyze interactions between cells in a model tumor microenvironment.

## 1. Introduction

Renal cell carcinoma (RCC) is among the ten most common cancers [1], and the most common histological subtype is clear cell renal cell carcinoma (ccRCC) [2]. Loss of the tumor suppressor *VHL* through genetic or epigenetic mechanisms is seen in over 90% of ccRCCs [3]. VHL is an essential component of the cellular oxygen sensor [4], and its loss induces a persistent pseudo-hypoxic state that results in a strong angiogenic profile of tumors [5]. Epithelial cells within the kidney cortex are thought to be the cells of origin for ccRCC, most likely proximal tubule epithelial cells [6]. Within the tumor, transformed epithelial cells are interspersed with a network of blood vessels, and the ccRCC stroma also contains interstitial fibroblasts and inflammatory and immune cells [7]. Cellular components of the tumor are embedded in the extracellular matrix (ECM).

The ECM environment controls many fundamental properties of tumors, including their proliferation [8,9], vascularization [10,11], and invasion [12], and is therefore a key determinant of their malignancy. The interplay between ECM and tumor cells is complex and multifactorial. In addition to providing the substrate for cancer cell attachment, ECM determines rigidity, which profoundly influences tumor malignancy [13]. Additionally, it directly controls signal transduction in cancer cells, which governs their behaviors [14]. The ECM is a complex mix of components [15], each with distinct physicochemical and signaling properties. Thus, the particular ECM composition characteristic of a tumor type is predicted to play an important role in determining the tumor’s behavior. Distinct tumors are predicted to differ in their ECM profiles, and this is likely a characteristic of the organ from which they arise as well as the tumor’s cellular composition, with cancer-associated fibroblasts (CAFs) acting as a major source of new tumor ECM [16]. CAFs are major contributors to the tumor ECM, but difficulties in isolating and maintaining these cells has limited their study. One important factor to the difficulties in culturing CAFs may be their dependence on the complex ECM environment that they generate [17]. Very little has been reported on CAFs in ccRCC, a tumor type with an extensive angiogenic and immunomodulatory stromal component. CAFs are lost over the course of a few weeks in cultures of dissociated ccRCC tumors encapsulated in collagen I [18], and mimicking the ECM from which these cells are isolated may be key to their longer-term maintenance.

Cell culture provides an essential tool for the study of human tumor biology, and cultured cells can be used as avatars of individual patient tumors for drug-response prediction [19]. Considering the strong influence of ECM on tumor cell biology, it is important that cultures replicate this component of tumors in addition to the cellular components. The matrix produced by the Engelbreth–Holm–Swarm (EHS) mouse sarcoma [20] known as Matrigel, Geltrex, or EHS matrix extract, is widely used to replicate the ECM environment for cultured tumor cells. The matrix isolated from this tumor is composed of three major components: laminin, collagen IV, and entactin/nidogen. It is used as the substrate to form organoids from both healthy and transformed intestinal tissue [21] and has been widely applied to the study of transformed tissues from other organs [22]. However, the composition of EHS matrix is similar to the ECM composition of healthy kidney cortex, leading us to question its utility in modeling ccRCC in vitro. We employed mass spectrometry to identify the major components of ccRCC ECM and found that in contrast to healthy kidney cortex, laminin, collagen IV, and entactin/nidogen are minor contributors. Instead, the tumor ECM is composed largely of collagen VI, fibronectin, and tenascin C. Analysis of single cell expression data indicates that CAFs may be a major source of tumor ECM production. Tumor cells as well as stromal cells bind efficiently to a nine-component ECM blend characteristic of ccRCC, whereas little binding is seen to Matrigel. Primary patient-derived tumor cells bind the nine-component blend efficiently, allowing us to establish mixed primary cultures with representation of tumor cells and stromal cells. These miniature patient-specific replicas are conducive to microscopy and can be used to analyze interactions between cells in a model tumor microenvironment.

## 2. Materials and Methods

### 2.1. Sample Collection

Kidney tumor tissue was obtained from surgical nephrectomies performed at the University of Pittsburgh Medical Center. Cancer diagnosis was performed by certified pathologists. Tissue adjacent to the surgical biopsy was collected at the discretion of the surgeon. All tissue was maintained in PBS at 4 °C and tested within 24 h of collection. All tissue specimens received were de-identified using an honest broker system. Demographical and pathological information provided for the nephrectomy specimens and whole kidneys may be found in Table 1.

### 2.2. Sample Processing

Samples were processed following the protocol from Pauli et al. [23]. Fresh tumor and adjacent normal tissue samples were diced and weighed. One hundred milligrams of tissue was flash frozen for Mass Spectrometry analysis, while remaining tissue was digested in 20× volume DMEM containing 250 U/mL Collagenase IV (ThermoFisher, Waltham, MA, USA) + 0.02% Trypsin-EDTA at 37 °C in a shaking incubator set to 200 RPM for up to 2 h or until media became cloudy. Digestion was stopped by adding an equal volume of ice-cold DMEM containing 10% FBS. Any undigested material was filtered with 100 µM filter and placed in a vessel containing 5× volume TrypLE Express (ThermoFisher) and returned to the 37 °C shaking incubator set to 220 RPM for an additional 15 min. Samples were pooled if necessary and centrifuged at 500× *g* for 7 min 30 s at 4 °C. Cells were washed with ice-cold DMEM and counted, and their viability was determined using Trypan Blue exclusion and utilized for downstream assays or frozen in Nutrifreeze (Sartorius, Göttingen, Germany) freezing media.

### 2.3. Proteomic Analysis

Peptide quantitative SWATH mass spectrometry was conducted as described elsewhere [24,25]. Proteins were prepared to provide approximately 100 µg total protein lysates. Proteins were reduced (DTT, 60 min at 30 °C) and then thiols were capped (iodoacetamide, 30 min at 30 °C) in 8 M urea. Urea was diluted to 1 M with Tris-HCl 8.0, and 1 µg sequencing grade trypsin was added per 30–40 µg protein and incubated overnight at 37 °C. Peptides were isolated on preparative C18 reverse-phase spin columns. LC for mass spec: 30 cm × 75 µm (inner diameter) high resolution C18 reverse phase chromatography. For proteomics informatics, an isotope-free unbiased scanning workflow, SWATH (Sequential Windowed Acquisition of All Theoretical Mass Spectra) [26,27] was used to allow isotope-free quantitation of proteins via LC/MS-based measurement of the levels of constituent tryptic peptides. A Triple-TOF 5600 (Sciex, Framingham, MA, USA) was programmed to acquire MS/MS data from as many as 100 variable retention time-widths, as determined by the LC trace of the proteome being analyzed. From these data, lists of proteins and their relative levels, with appropriate statistics (e.g., *p*-values < 0.05, false discovery rate, FDR < 1%) were provided using a software workflow that is comprised of Sciex proprietary programs; ProteinPilot (data-dependent peptide ion libraries), PeakView (SWATH app to link SWATH data and ion library data, quantify peaks, provide FDR and quality control analytics), and MarkerView (principle component visualization [28] and statistical analysis of group comparisons). Additional detail may be found in Supplemental Methods. SWATH and ion library raw data will be located at the PeptideAtlas repository and accessible via the web. PeptideAtlas is a part of the ProteomeXchange Consortium.

### 2.4. Pathway Analysis

Processed SWATH data were functionally annotated using DAVID, Gene Set Enrichment Analysis (GSEA), and Gene Ontology Analysis (GO) using only proteins showing significant differential expression (*p*-value < 0.05). Exported data can be found in Tables S2–S4. Additional detail may be found in Supplemental Methods.

### 2.5. Kidney Tumor ECM Analysis

Processed Mass Spectrometry data were further annotated and sorted using definitions from matrisomeDB [29,30] and can be found in Table S1. Samples were grouped and analyzed based on origin, either tumor or adjacent normal kidney. ECM components containing the gene ontology term GO:0050817 (coagulation) were labeled and excluded from this analysis. SWATH and DIA datasets were combined and annotated for differential expression (*p*-value < 0.05) and relative total protein abundance (%LFQ). Highly abundant core matricellular proteins (>0.1% LFQ), were renormalized to total percent core matrisome and statistically characterized. The most abundant targets (% ≧ Quartile 3) were used in the construction of “ccRCC ECM” blend. 

### 2.6. Flow Cytometry

Digested tumor or adjacent normal cells were centrifuged and resuspended at 5 × 10^5^–2 × 10^6^ cells/100 µL FACs buffer (Miltenyi, Bergisch Gladbach, Germany). Cells were divided into no antibody control, viability analysis panel (Propidium iodide, Hoeschst33342, DRAQ7), IgG control (REA-VioBlue, REA-FITC, REA-PE, REA-APC, REA-PE-Vio770), staining panel 1 (1:50 CD31-VioBlue (Miltenyi 130-117-227), 1:11 CA9-PE (Miltenyi 130-110-057), 1:10 PDGFRβ-APC (Miltenyi 130-105-322), 1:10 PDGFRα-APC (Miltenyi 130-115-239), 1:50 CD326-PE-Vio770 (Miltenyi 130-111-002), 1:50 CD45-FITC (Miltenyi 130-110-631), Propidium Iodide), or staining panel 2 (1:50 CD10-VioBlue (Miltenyi 130-114-509), 1:11 CA9-PE, 1:50 CD105-PE-Vio770 (Miltenyi 130-112-167), CD184-APC (Miltenyi 130-098-357), 1:50 CD326-PE-Vio770, Propidium Iodide), and incubated in the dark at 4 °C for 15 min. Cells were washed two times with flow buffer and analyzed using Miltenyi AutoMACs flow cytometer. Side and forward scatter gating were determined using viability analysis panel to identify cells vs. debris. Doublets were excluded using SSR-H versus SSR-L. Positive gating for each marker was determined using IgG control. Compensation was performed using the MACS Comp Bead kit, anti-REA (Miltenyi 130-104-693).

### 2.7. Primary Cell Culture

Adapted from Williams et.al [31]. Digested tumor cells were washed and resuspended in 1 × 10^6^ cells/5 mL High Glucose DMEM containing 10% FBS, 1% GlutaMax (ThermoFisher), 1% Penicillin-Streptomycin, 1% Non-Essential Amino Acids (ThermoFisher), and 1% Sodium Pyruvate and plated onto 5 cm tissue culture treated plates. Plates were incubated at 37 °C for 24 h before media change and imaging. Cells were monitored with media change every 72 h. After reaching 70–80% confluency, cells were expanded into a 10 cm tissue culture treated dish. Upon reaching 70–80% confluency, passaged cells were either frozen in FBS/DMSO freeze media or further expanded for downstream applications.

### 2.8. Cell Line Culture

All cells were obtained from ATCC, Manassas, VA, USA. 786-O (ATCC CRL-1932), NRK-49F (ATCC CRL-1570), MS1 (ATCC CRL-2279), and RAW264.7 (ATCC TIB-71) cells were maintained or adapted to RPMI-1640 containing 10% FBS, 1% GlutaMax (ThermoFisher), and 1% Penicillin-Streptomycin and grown to 70–80% confluency on tissue culture treated plates before experiments. Cells were detached from plates using TrypLE Express (ThermoFisher) with the exception of RAW264.7 cells, which required use of a cell lifter.

### 2.9. Immunocytochemistry Validation of Primary Cell Lines

Upon freeze or expansion of primary cell cultures, 5000 cells in 500 µL of culture media were transferred into a Cytofunnel and centrifuged for 5 min at 1000 RPM in a Shandon CytoSpin centrifuge (ThermoFisher cat#A78300003). Slides were dried and frozen at −70 °C. Slides were brought to room temperature submerged in 4% PFA for 15 min. Slides were washed before permeabilization with 0.02% TritonX-100 in PBS for 10 min. Blocking was done using 5% Donkey Serum in PBST for 1 h at room temperature. Primary antibody staining was done at 4 °C overnight with the following antibodies and dilutions: Vimentin 1:400 (MilliporeSigma, Burlington, MA, USA V6630), Cytokeratin 18 (R&D, Minneapolis, MN, USA AF7619), and PDGFRα/β 1:100 (abcam, Cambridge, UK ab32570). Secondary staining was done alongside DAPI using the following antibodies: Donkey anti-Mouse AlexaFluor 647 (ThermoFisher A31571), Donkey anti-Rabbit AlexaFluor 568 (ThermoFisher A10042), and Donkey anti-Sheep AlexaFluor 488 (ThermoFisher A11015). Slides were mounted in Vectashield (Vector, Burlingame, CA, USA) and imaged on a Leica DMI 6000B. Tumor cells were identified as VIM^+^CK18^+^PDGFRα/β^−^, while fibroblasts were defined as VIM^+^CK18^−^PDGFRα/β^+^. Analysis was repeated for three primary tumor lines at passages 1, 2, and 4.

### 2.10. Cell Line Attachment Assays

One day in advance, sterile non-tissue culture treated 96-well plates were coated with ECM substrates at a concentration of 2 µg/mL in sodium carbonate/bicarbonate binding buffer for 2 h at 37 °C. Matrigel coating was created by dilution to 8 µg/mL in cell culture media. Plates were then transferred to 4 °C to incubate overnight. Uncoated wells were incubated with just binding buffer. ECM coating solution was aspirated before preparing cells and left to air dry in the back of the tissue culture hood. The following proteins were used for this and all other experiments: Collagen VI (Corning, Corning, NY, USA 8064002), HSPG2 (R&D 2364-ER), Laminin (MilliporeSigma L6264), Tenascin-C (R&D 3358-TC-050) Fibronectin (MilliporeSigma FC010), Fibrillin-1 (R&D 10224-F1-050), Lumican (R&D 2846-LV), Collagen 12 (Novus, Uppsala, Sweden NBP1-88062PEP), Periostin (R&D 3548-F2-050), and TGFBI (R&D, 3409-BG-050). Single cell suspensions were diluted to 20 cells/µL in assay media (RPMI-1640 without FBS), and 100 µL were added to each well of coated or uncoated 96-well plates. Plates were incubated at 37 °C in a cell culture incubator. After 2 h, wells were aspirated and briefly rinsed with DPBS. Wells were imaged using EVOS microscope (ThermoFisher). Cell attachment was determined by morphological characteristics such as cell spreading, elongation, and presence of projections.

### 2.11. Primary Cell ECM Attachment and Growth

Eight-well Permanox chamber slides (Nunc, Rochester, NY, USA) were coated with ccRCC ECM blend or DPBS only and incubated for 2 h at 37 °C. Plates were then transferred to 4 °C to incubate overnight. Prior to cell seeding, chambers were aspirated and dried in back of tissue culture hood. Primary cell digests were diluted in DMEM without FBS and cultured up to 4 h at 37 °C. Chambers were then either rinsed and fixed in 4% PFA for 15 min or replaced with DMEM + 10% FBS culture media for an additional culture period of 72 h before fixation. Fixed chambers were rinsed with PBS before permeabilization with 0.02% TritonX-100 in PBS for 10 min. Blocking was done using 5% Donkey Serum in PBST for 1 h at room temperature. Primary antibody staining was performed overnight at 4 °C against the following targets; PDGFRα/β 1:100 (abcam ab32570), CD45 1:100 (Novus NBP2-80652), CD34 (BioRad, Hercules, CA, USA, MCA547G), PAX2/8 1:200 (ThermoFisher 71-6000, ProteinTech, Rosemont, IL, USA 10336-1-AP), Vimentin 1:1000 (MilliporeSigma AB5733), CK18 (R&D AF7619), and CXCR4 1:100 (abcam ab124824). After washing, secondary antibody staining alongside DAPI was performed for 1 h at room temperature with the following antibodies: Donkey anti-Rabbit AlexaFluor 568 (ThermoFisher A10042), Donkey anti-Chicken AlexaFluor 488 (abcam ab150169), Donkey anti-Mouse AlexaFluor 647 (ThermoFisher A-31571), Donkey anti-Rat AlexaFluor 647 (ThermoFisher A-21247), and Donkey anti-Sheep AlexaFluor 488 (ThermoFisher A11015). Mounting in EverBrite (Biotium, Fremont, CA, USA cat#23018). Imaging was performed using Leica Thunder Imager. Images were deconvolved using the Leica Thunder small volume processing algorithm and analyzed using LASX software. Fibroblasts were defined as PDGFRα/β ^+^CD45^−^Vim^+^ and negative for all carcinoma markers. Endothelial cells were defined as CD34^+^CD45^−^ and negative for all carcinoma markers. Immune cells were defined as CD45^+^CXCR4^+/−^ and negative for remaining carcinoma markers_._ Tumor cells were defined as PDGFRα/β^−^CD45^−^CD34^−^ and positive for any two of the following carcinoma markers: CK18, VIM, PAX2/8, and CXCR4. All other cells were categorized as “Other”. Distribution of cell types was clustered using ClustVis webtool (https://biit.cs.ut.ee/clustvis/ (accessed on 6 January 2021)) using Pareto row scaling. For EdU incorporation analysis, EdU was added to culture media at final concentration of 5 µM and added to wells of 4 h after attachment. Cells were cultured for up to three weeks, at which chambers were fixed in 4% PFA and immunostained as previously summarized. EdU was detected using the Click-iT EdU Cell Proliferation Kit (ThermoFisher) standard protocol. Imaging was conducted as stated above.

### 2.12. D Cultures

Fibrin domes were created following modifications to protocol by Liu et al. [32]. In summary, fibrin domes were generated by combining fibrinogen (MilliporeSigma cat#F8630) at a 1:1 ratio with cells suspended in culture media with or without addition of ECM proteins. Cell concentration was cell-line-dependent following testing; 786-O = 50 cells/µL, S108 = 150 cells/µL, S109 = 150 cells/µL, S114 = 100 cells/µL, Primary Tissue Digests = 200 cells/µL. Thrombin (MilliporeSigma cat#10602370001) at a concentration of 0.01 U/µL was added at a volume equal to 10% of total to cell–fibrinogen mix, then immediately spotted onto glass coverslips at 5 µL per replicate. Domes were immediately placed into a 37 °C cell culture incubator for 10 min to facilitate gelation. Pre-warmed cell culture media (786-O: RPMI-1640 containing 10% FBS, 1% GlutaMax (ThermoFisher), and 1% Penicillin-Streptomycin; primary cultures and tissue digests: DMEM containing 10% FBS, 1% GlutaMax, 1% NEAA (ThermoFisher), and 1% Penicillin-Streptomycin, or CHM obtained from Weill Cornell Englander Institute for Precision Medicine [23]) were carefully added to wells and allowed to culture in cell culture incubator. Media were changed every 2–3 days. Matrigel domes were created following modification of protocol by Sato et al. [21]. In summary, Matrigel domes were generated by combining Matrigel GFR (Corning cat#354230) at a 1:1 ratio with cell suspended in culture media at before mentioned concentrations, then immediately spotted onto glass coverslips at 5 µL per replicate. Domes were immediately placed into a 37 °C cell culture incubator for 30 min to facilitate gelation. Pre-warmed cell culture media were carefully added to wells and allowed to culture in cell culture incubator. Media were changed every 2–3 days.

### 2.13. Live/Dead and Structure Analysis

At designated end-points (786-O = 6 days, Primary ccRCC Tumor Lines = 30 days, Primary Tumor Digests = 19 days), media were replaced with Live/Dead staining solution containing 2 μM Calcein-AM and 4 μM Ethidium homodimer-2 (Ethd2) in culture media (ThermoFisher cat#L3224) and incubated for 30 min in culture incubator. In the last remaining 8 min, Hoechst 33,342 (ThermoFisher cat# H3570) at a final concentration of 5 µg/mL was added to each sample. Three-dimensional domes were washed 3 × with DPBS and mounted for fluorescent imaging in phenol-free media. Collected images were deconvolved using Leica LasX Thunder deconvolution algorithm. Deconvolved images were analyzed for Hoechst 33,342 positivity to identify number of cellular structures. Each structure was designated as either live (Calcein^+^) or dead (Ethd2^+^). Comparisons between culture conditions were done via Students’ *t*-test.

### 2.14. D Dome Immunostaining

3D cultures were fixed in 4% PFA for 15 min at room temperature and washed 3x with PBS. Cell permeabilization was performed using 0.2% TritonX-100 in PBS for 15 min. Blocking was performed using 5% Donkey Serum (Jackson Immunoresearch, West Grove, PA, USA, cat# 017-000-121) in PBST for 1 h. Primary antibody incubation against listed targets was done by incubating overnight at 4 °C at associated dilutions in PBST: PDGFRα/β 1:100 (abcam ab32570), CD31 1:200 (ThermoFisher MA3100), Vimentin 1:1000 (MilliporeSigma AB5733), Renin 1:100 (abcam ab212197), CD45 1:100 (Novus NBP2-80652), ACTA2 1:50 (MilliporeSigma A5228), and CXCR4 1:100 (abcam ab124824). Rigorous washing was performed the next day for 6 h before secondary incubation with DAPI and following antibodies at 1:250 dilution in PBST at 4 °C overnight: Donkey anti-Rabbit AlexaFluor 568 (ThermoFisher A10042), Donkey anti-Chicken AlexaFluor 488 (abcam ab150169), Donkey anti-Mouse AlexaFluor 647 (ThermoFisher A-31571), and Donkey anti-Rat AlexaFluor 647 (ThermoFisher A-21247). Rigorous washing was performed the next day for 6 h, followed by an additional overnight wash at 4 °C overnight. Sample clearing was performed in series moving from 25%, 50%, and 80% glycerol every 30 min before mounting in EverBrite (Biotium cat#23018). Imaging was performed using Leica Thunder Imager. Selected images for publication were deconvolved using Leica LasX Thunder deconvolution algorithm.

### 2.15. EdU Incorporation and Detection

EdU was added to culture media at final concentration of 5 µM and maintained for 48 h. Domes were then fixed and immunostained following above procedure above. EdU was detected using the Click-iT EdU Cell Proliferation Kit (ThermoFisher) standard protocol. Imaging was conducted as stated above.

### 2.16. scRNA-seq Analysis

Publicly available scRNA-seq data from Young et al. 2018 (European Genome-Phenome Archive study IDs EGAS00001002171, EGAS00001002486, EGAS00001002325 and EGAS00001002553) was downloaded from BBrowser and used for analysis [33]. Plots were generated using t-SNE, and clusters were generated by unsupervised k-means. Identification of clusters was determined through the normalized expression of chosen cell identity markers (Fibroblasts: PDGFRβ, PDGFRα; Epithelia: AQP1, AQP2, EPCAM; Endothelia: PLVAP, CD31, CD34; Immune: CD45; Cancer; CXCR4, VIM, KRT18, PAX8, PAX2, CA9, CD10), in which a cluster presented higher than average expression levels of one or multiple cell fate markers and exclusion of other fate determining markers. After manual categorization of clusters, expression of identified ECM markers was scored across all clusters based of normalized expression (above average = +, 2 × above average = ++, >2 × above average = +++). Comparisons between clusters was done via differential expression analysis of transcript counts and weighted by log fold change. Data can be found in Tables S5 and S6.

### 2.17. ProteinAtlas Analysis

Identified ECM proteins were investigated for expression pattern from immunostained tissue on ProteinAtlas [34]. If multiple antibodies were available for reference, we chose the one with the highest level of validation (“Enhanced”). We included or eliminated ECM markers from further analysis if they met the following criteria: A. If present, had only an interstitial staining pattern in tumors; B. If present, had only an interstitial staining pattern in normal kidney; and C. If present, did not have staining in the glomeruli of normal kidney. This is summarized in Figure S15.

### 2.18. Immunohistochemistry

Immunostaining of serial sections was conducted as previously described [35] on paraffin-embedded ccRCC tumor sections. Primary antibody PDGFRα/β 1:100 (abcam ab32570) or ACTA2 1:50 (MilliporeSigma A5228) and biotinylated goat anti-rabbit 1:500 (Vector BA-1000) were used for immunohistochemistry using Vectastain ABC Elite kit (Vector PK-7100), and the color reaction was performed using DAB. Imaging was performed on a Zeiss Axioskop 2.

## 3. Results

### 3.1. Isolation and Culture of Cells from ccRCC Tumors Using Standard Methods

**Figure 1:**
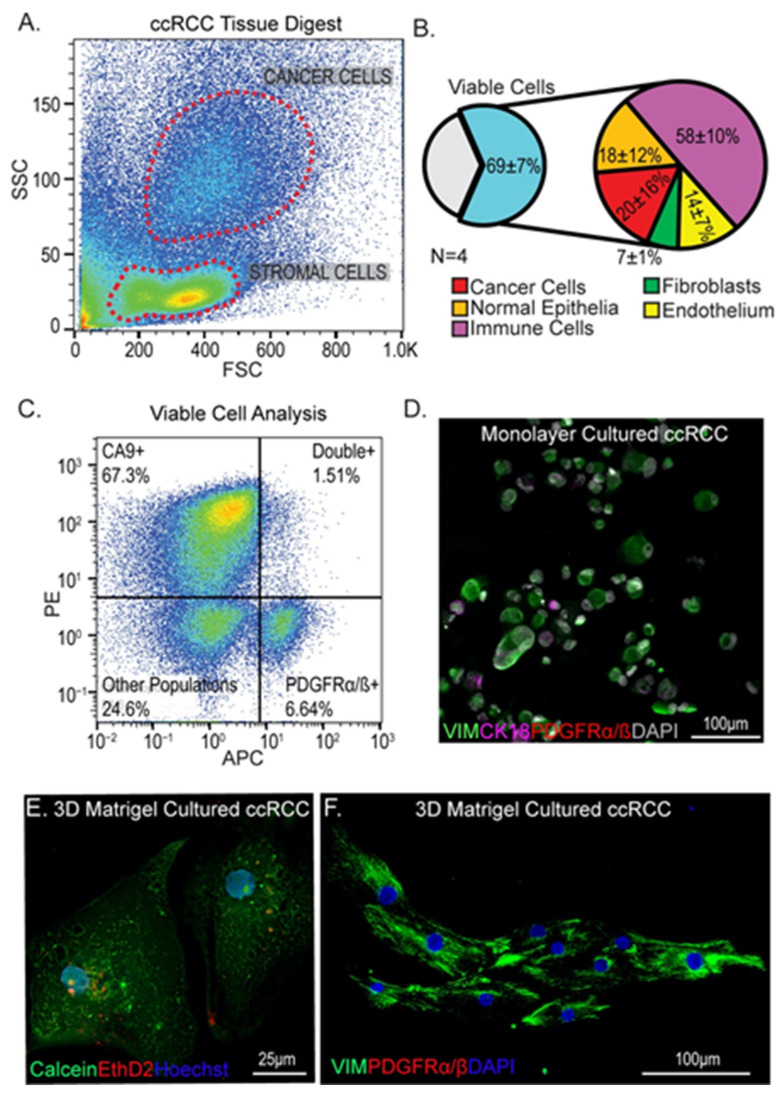
Identification and culture of fibroblasts from ccRCC tumors. (**A**) Representative flow cytometry cell marker analysis of ccRCC tumors can allow distinction of stromal populations (low-side scatter) from cancer cells (high side scatter). (**B**) Summary of marker analysis of different cell types from four different patient tumors. Viability determined by propidium iodide exclusion. (**C**) Identification of fibroblasts using PDGFRα/β, showing distinction from CA9+ tumor cells. (**D**) Standard monolayer culture of isolated tumor cells showing lack of PDGFRα/β+ stromal cells. Green = vimentin, magenta = CK18, red = PDGFRα/β, gray = DAPI. (**E**,**F**) Subsets of cells from primary tumor digests are viable when cultured in Matrigel domes (**E**) but no evidence of PDGFRα/β + stromal cells could be found (**F**). Green = vimentin, red = PDGFRα/β, blue = DAPI.

To understand the representation of cell types in cultures of ccRCCs, we dissociated four ccRCC tumor samples using previously published methods [23] and analyzed cells by flow cytometry (Figure 1A and Figure S1). Using a panel of markers for tumor cells (CA9 and CD10), immune cells (CD45), endothelial cells (CD31), epithelia (CD326), and fibroblasts (PGFRα/β^+^), we found significant representation of viable cells, determined via propidium iodide exclusion, corresponding to all of these different cell types (Figure 1A,B and Figure S1). In particular we were interested to see that approximately 7% of cells isolated from tumors were PGFRα/β^+^ putative fibroblasts (Figure 1B,C). Using a standard protocol for monolayer tumor cell growth on tissue-culture-treated plastic in serum-containing medium [31], primary cell cultures were established and stained for tumor (CK18, vimentin) and fibroblast (PDGFRα/β) cell markers. PDGFRα/β ^+^ cells were poorly represented in these monolayer cultures; initially, the proportion of PDGFRα/β^+^ cells plated was 15.04 ± 3.76%, but after outgrowth the percentage was only 3.22 ± 0.39% (Figure 1D and Figure S2). The discrepancy between the flow analysis (7% of cells PGFRα/β ^+^) and first passage of culture (15% of cells PGFRα/β ^+^) indicates that other cell types may also be disadvantaged in this culture system. One explanation for the paucity of fibroblasts seen after outgrowth of primary cells may be a lack of attachment; in establishment of monolayer cultures from primary tumor isolates, a substantial proportion of cells remained unattached 48 h after plating. However, the small number of fibroblasts that did grow out displayed a rounded morphology rather than the characteristic elongated morphology of fibroblasts, and they were outcompeted after serial passaging (the proportion at passage 5 was 0.55 ± 0.15%) (Figure S2). This was unanticipated since the culture conditions included 10% fetal bovine serum, which is reported to provide a potent growth advantage to fibroblasts in primary cell culture [36,37]. To ask if we could use a three-dimensional (3D) culture system to improve the representation of tumor fibroblasts, we seeded cells into Matrigel domes using standard tumor organoid procedures [21]. While viable cells were detected in the Matrigel (Figure 1E), no PDGFRα/β ^+^ fibroblasts could be found after 19 days of culture (Figure 1F), demonstrating that these conditions further disadvantaged fibroblast growth. Based on these findings, we concluded that primary ccRCC-derived fibroblasts are unexpectedly fastidious in their culture requirements and may require growth conditions that more accurately reflect the tumor microenvironment. The ECM is a key component of the microenvironment that strongly influences cell behavior, which led us to investigate the composition of ccRCC ECM.

### 3.2. Differences in ECM Composition between ccRCC and Neighboring Healthy Cortex

**Figure 2:**
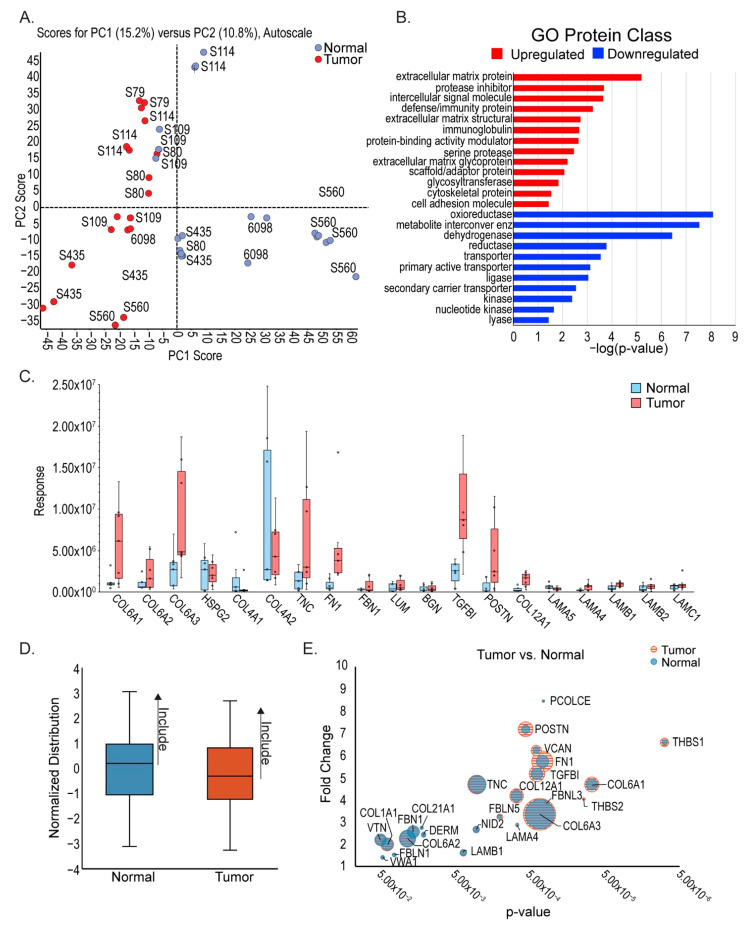
ECM protein upregulation in ccRCC. (**A**) Principal component analysis of mass spectrometry data from stage 3 ccRCC tumors and matched adjacent healthy cortex shows separation between tumor and normal. (**B**) Gene Ontology (GO) analysis of identified proteins shows highest upregulation of the protein class PC00102: extracellular matrix protein. (**C**) Analysis of highest upregulated and downregulated ECM proteins identified by mass spectrometry data and cross-referenced with the Matrisome database (MatrisomedDB). (**D**) Summary of DDA mass spectrometry analysis, indicating inclusion of only the most abundant ECM proteins. (**E**) Combined SWATH and DDA analyses to visually represent most significant ECM protein changes between tumor and adjacent normal kidney.

Considering its major constituents, Matrigel quite accurately represents epithelial basement membrane, and we hypothesized that cells isolated from tumors may require a tumor-matched ECM in order to grow out in proportions representative of the tumor of origin. To compare ECM composition between ccRCC tumor and healthy neighboring cortex, we performed mass spectrometry analysis of patient tumor samples. Kidney tumor samples were obtained from surgical nephrectomies, stored on ice at the time of surgery, dissected, and flash frozen within 24 h of collection. Tumors with healthy margins used in this analysis were all stage 3, as determined by a pathologist, and are listed in Table 1. Exploratory SWATH mass spectrometry, conducted using a kidney-sample-derived ion library [38], as previously described [24,25], was used for this analysis since previous studies have demonstrated its utility in quantitative profiling of the matrisome in unenriched tissues [39]. Unsupervised clustering of normalized proteomic data, presented as a principal component analysis (PCA) [28], shows separation between the tumor and adjacent normal cortex tissue primarily on the first principal component (Figure 2A).

SWATH analysis provided relative protein differences between grouped stage 3 ccRCC tumors and adjacent healthy kidney cortex using Sciex MarkerView (Table S1). To determine which biological pathways differ between ccRCC and neighboring healthy cortex, we performed Gene Ontology (GO) analysis (Figure 2B, Table S2). Mitochondrial metabolism, including oxireductases, metabolite interconversion enzymes, and dehydrogenases, were the most downregulated pathways. This provides a degree of confirmation of our approach since these pathways are predicted to be downregulated in ccRCC due to the heavily glycolytic profile of the tumor [40]. Interestingly, the most significant upregulated class was PC00102: Extracellular Matrix. Confirmatory analyses using GSEA and DAVID were performed, showing similar results such as clustering of pathways related to ECM signaling (Figure S3A, Table S3) and significant enrichment of ECM (Figure S3B, Table S4), respectively.

To define the specific differences in ECM composition between ccRCC and neighboring healthy cortex, we used the matrisome database MatrisomeDB [29] to identify ECM proteins from each proteomic profile. Since we were interested in structural changes between ccRCC and the healthy cortex, we narrowed our analysis to components of the “kidney cortex matrisome”, i.e., glycoproteins, collagens, and proteoglycans [41]. Substantial differences between tumor and neighboring healthy cortex were found in both interstitial matrix proteins, such as collagen 6 (COL6A1, COL6A2, COL6A3), and basement membrane components such as collagen 4 (COL4A1, COL4A2) and laminins (LAMA5, LAMA4, LAMB1, LAMB2, LAMC1) (Figure 2C). While it revealed significant relative changes in protein abundance, SWATH analysis did not have the sensitivity to accurately measure the abundance of each protein. Using the same samples, we performed data-dependent acquisition (DDA) mass spectrometry, selecting for the 50 most intense ions. Due to its superior sensitivity, a DDA approach allowed for accurate quantitation of ECM proteins using Maxquant [42]. The most abundant proteins, based on a normal distribution, were selected and segregated for further analysis (Figure 2D). Using both the SWATH and DDA data, a combined dataset was produced that compares both the differential expression and quantity of proteins between stage 3 ccRCC tumors and adjacent health cortex (Figure 2E, Table S1).

### 3.3. Transcriptional Analysis Indicates That Tumor Fibroblasts Are Major Contributors to ccRCC ECM

**Figure 3:**
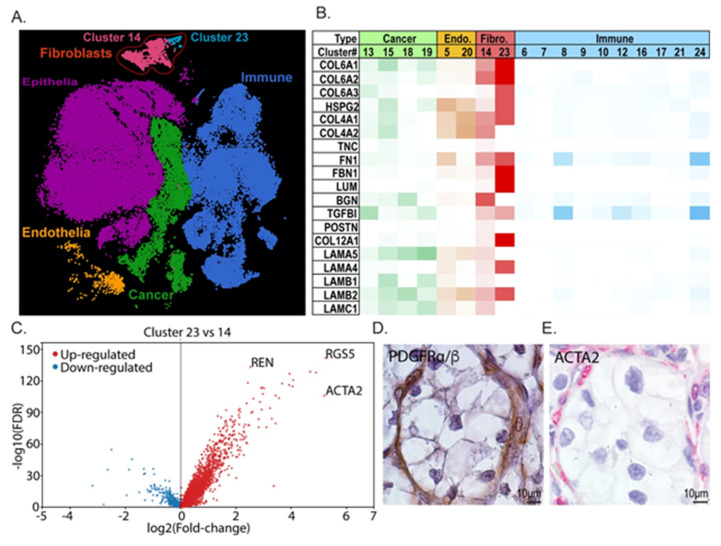
Prediction of cellular sources of matrix molecules based on scRNA-seq. (**A**) scRNA-seq analysis of ccRCC tumors from Young et al. [33]. Unbiased clustering was performed, and clusters were identified using cell-type-representative transcripts. Distinct fibroblast subclusters are indicated and outlined in red. (**B**) Differential ECM gene expression analysis of clusters identified as discrete cell populations. Endo. = endothelial cells, Fibro. = fibroblast cells. (**C**) Comparative transcript analysis between fibroblast sub-clusters 23 and 14 shows differential expression of cancer associated fibroblast markers. (**D**,**E**) Representative IHC from patient ccRCCs for PDGFRα/β^+^ (**D**).

Stromal populations are commonly associated with tumor ECM remodeling and contribute significantly to tumor growth [43,44,45]. To determine if these cells could be the source of ECM in ccRCCs, we analyzed scRNA-seq data from Young et al. [33] for transcripts related to the top ECM targets we identified. First, we categorized clustered data into tumor and stromal populations using a panel of markers for each (epithelial: AQP1, AQP2, EPCAM; endothelial: PLVAP, CD31, CD34; fibroblast: PDGFRα, PDGFRβ; immune: CD45; tumor cell: CXCR4, VIM, KRT18, PAX8, PAX2, CA9, CD10) (Figure 3A, Table S4). Then, we stratified the expression of each ECM component for mean expression and scored clusters for high and low expression (Figure 3B). Two clusters of cells were identified as fibroblasts, and interestingly, these showed the highest expression of a number of genes encoding ECM proteins that are abundant in ccRCC such as collagen VI isoforms, fibronectin, lumican, and collagen XII. Fibroblast cluster 23 was the most predominant ECM-expressing subset of all cells in the analysis. We hypothesized that this subpopulation of PDGFRα/β^+^ fibroblasts may be a cancer-associated fibroblast (CAF) population, and we analyzed it further to determine if it expressed a characteristic transcriptome profile. ACTA2, IGFBP7, TAGLN, MYL9, and MYLK are significantly overexpressed in cluster 23 compared with other PDGFRα/β^+^ cells, suggesting that this subpopulation may indeed be a ccRCC CAF population (Figure 3C, Table S6) [46,47,48].

To spatially identify putative CAFs within tumor tissue, we immunostained patient tumors with PDGFRα/β to label fibroblasts and pericytes, and ACTA2 to label activated tumor fibroblasts (Figure 3D,E). Reflecting the single cell data, there is an extensive PDGFRα/β population within the stroma of the tumor tissue (Figure 3D). The ACTA2-expressing population is a small subset of these cells that is disseminated within the stroma (Figure 3E). The scRNA-seq analysis also revealed that the ACTA2-high putative CAF population shows high expression of REN (Figure 3C, Table S6), suggesting that it overlaps with the renin-producing cell population identified within the ccRCC stroma by other investigators [49].

### 3.4. Binding of Tumor and Stromal Cells to ccRCC ECM Components

**Figure 4:**
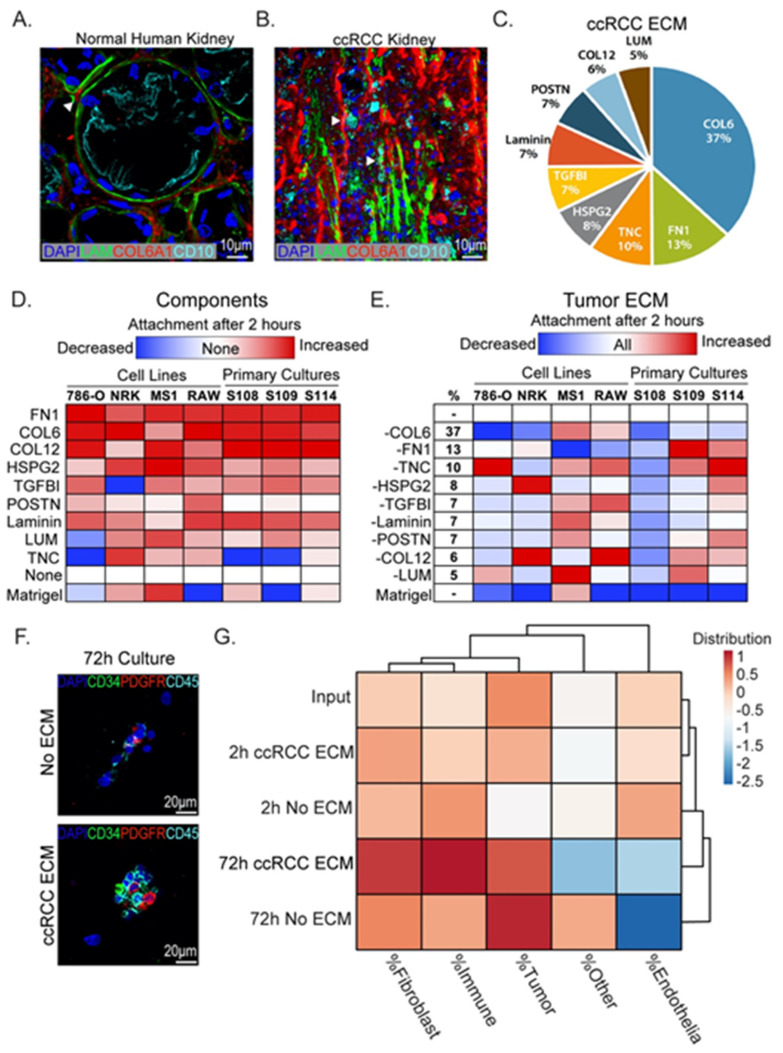
ECM effects on tumor and stromal cell attachment. (**A**) Immunofluorescence staining of healthy human kidney showing structured tubule basement membrane separating CD10+ proximal tubule epithelium from interstitial collagens (white arrowheads). Blue = DAPI, green = laminin, Red = collagen 6 alpha 1, cyan = CD10. (**B**) Immunofluorescence staining of ccRCC tumor for ECM components showing loss of the tubule basement membrane and mixing of laminin and interstitial collagens (white arrowheads). Blue = DAPI, green = laminin, red = collagen 6 alpha 1, cyan = CD10. (**C**) Pie chart summarizing major components of ccRCC ECM from mass spectrometry analysis. (**D**) Analysis of attachment of cell cultures to ECM components identified by mass spectrometry. Cell attachment to 2 µg/mL of indicated ECM-coated wells was determined after 2 h. Heatmap indicates increased cell attachment (red) and decreased attachment (blue) relative to no coating condition (“None”). (**E**) Cell attachment to coated dishes in the presence of all proteins or lacking one component, indicated by “-” sign. Analysis was done relative to complete blend condition “All”. Total percentage each individual component takes up in the total blend (2 µg/mL total protein) is indicated in the “%” column. (**F**) Freshly digested tumor tissue 72 h after plating on ccRCC ECM or noncoated plates. Scale bar = 20 µm. Blue = DAPI, green = CD34, red = PDGFRα/β, cyan = CD45. (**G**) Cluster analysis comparing cultures grown on coated or uncoated chamber slides to initial cell input.

In healthy tissue of the cortex, ECM is organized into basal lamina on which epithelial cells sit, and interstitial ECM, which provides structural integrity and conduits for vasculature and nerves. A representative section from adjacent healthy cortex stained for the basal lamina component laminin and the interstitial component collagen VI is shown in Figure 4A. Healthy proximal tubule epithelia are characterized by basal localization of laminin, which separates them from interstitial ECM components. In contrast, ccRCC tissue shows a breakdown of this stereotypical arrangement with mixing of basal lamina and interstitial ECM components (Figure 4B). Based on this analysis, we conclude that cells within tumors are exposed to a mixture of basal lamina and interstitial ECM components, in contrast to healthy proximal tubule epithelial cells, which are only exposed to the basal lamina. Using the relative abundances of ECM proteins characterized in our proteomic analysis, we generated an ECM blend characteristic for ccRCC (Figure 4C). This blend is based on the nine most abundant components identified in our analysis and includes approximately 80% of all components identified in tumor ECM (Table S1). To define the binding profiles of tumor and stromal cells to individual proteins that are most abundant in tumor ECM, we measured the binding capacity of cell lines representative for each cell type to ECM components using a monolayer attachment assay. Tumor cells (786-O), fibroblasts (NRK-49F), endothelial cells (MS1), and macrophages (RAW 264.7, abbreviated to RAW) were used to represent the common cell populations found in tumors. Single cell suspensions were given 2 h to attach to ECM-coated or uncoated wells before quantification of bound cells (Figure S4). This time-point was chosen as it precedes non-specific attachment of cells to uncoated polystyrene (data not shown). As a comparator we included Matrigel, which is commonly used to model tumor ECM and enhance attachment of tumor cells. Attachment to Matrigel was seen with endothelial cells and fibroblasts, while tumor cells and macrophages attached poorly. In contrast, all cell types attached efficiently to the two most abundant ECM molecules that we identified in tumors by mass spectrometry: collagen VI and fibronectin. We found that the cell lines analyzed shared common preferences to our chosen ECM components (Figure 4D). Fibronectin (FN1), collagen VI (COL6), collagen XII (COL12), and heparan sulfate proteoglycan 2/perlecan (HSPG2) showed the strongest binding across all cell types. Some components repelled certain cell types, for example, TGFBI, which decreased cell attachment of fibroblasts, and lumican and tenascin C, which reduced attachment of tumor cells.

To validate if these findings could be applied more broadly to ccRCC tumor cells, we isolated primary tumor cell cultures from three patient tumors as described in [23]. Cell lines S108, S109, and S114 express characteristic markers of ccRCC tumor cells but show distinct morphologies (Figure S5). The cell binding study was repeated using these primary tumor cell lines and showed a binding profile very similar to 786-O, with strong binding to fibronectin, collagen VI, and collagen XII and inefficient binding to or repulsion by periostin, lumican, and tenascin C (Figure 4D). Results from these experiments suggest that a core group of ECM molecules promotes attachment of diverse ccRCC tumor cells.

We were interested to understand if there could be synergistic activities between ECM molecules identified in our study, and we next asked how the effects of the single components would compare to a combination of all molecules in our ECM blend. We found that all cell types adhered more efficiently to our ccRCC ECM blend compared to Matrigel with the exception of endothelial cells (Figure 4E). To understand the contribution of each component of the blend, we subtracted them individually and compared the binding efficiency of cells to the complete blend (Figure 4E). Although removal of certain components could increase the binding efficiency of individual populations, removal of no one individual component promoted attachment of all.

### 3.5. ccRCC ECM Blend Binds Diverse Cell Types Isolated from Patient Tumors

Based on the results of our cell binding experiments with immortalized and primary cell lines, we predicted that the ccRCC ECM blend could capture discrete cell populations directly from patient tumors. Utilizing previously published techniques [23], we dispersed ccRCC patient tumor samples into single cell suspensions, which we gave 2 to 72 h to attach to ECM-coated or uncoated chamber slides. Due to the poor attachment of cells to Matrigel, we did not include this coating as a comparator. The following markers were used to identify stromal cell populations: fibroblasts (PDGFRα/β), endothelia (CD34), and immune cells (CD45) (Figure 4F). At each time point analyzed, the percentage of each cell type was compared to the percentage of original input cells determined from a smear of the cell suspension isolated from each tumor. Cluster analysis based on cell composition was performed to identify the culture condition that best matched the repertoire of original input cells derived from the tumor (Figure 4G). While showing an increased preference for fibroblasts and tumor cells, the ccRCC ECM blend after 2 h was the closest match to the input. Following 72 h, while no longer accurately reflecting the initial input, the ccRCC ECM blend better maintained the distribution between fibroblast and immune cells when compared to no ECM. Additionally, endothelial cells were also better maintained, although at reduced abundance compared to earlier time points. To understand if the change in cell distribution after 72 h was due to differences in cell growth, primary cell mixes were cultured on ccRCC ECM-coated chamber slides in the presence of the thymidine analog EdU, which is incorporated into DNA of cycling cells. EdU was supplemented for the first week of culture (“pulse”) and subsequently removed for one week (“chase”) to label dividing cells. Two out of three samples analyzed had viable cells by the end of this time course. Although the distribution of fibroblasts was better maintained on ccRCC ECM compared to no ECM, little EdU labeling was found on these cells, indicating that ccRCC ECM promoted survival but not proliferation (Figure S6).

In conclusion, the use of ccRCC ECM can improve the capture of unique cell populations from a tumor sample and can better maintain different cell identities from 2 to 72 h when compared to conventional monolayer tissue culture conditions. However, the monolayer culture system was unable to promote outgrowth of fibroblast populations, regardless of ECM coating.

### 3.6. Design of a 3D Model of ccRCC with Native ECM Environment

Although the inclusion of ccRCC ECM in monolayer culture showed improved cell representation from tumors over Matrigel- and non-coated conditions, poor viability after extended culture was problematic. The use of 3D organoid culture systems for in vitro tumor modeling has gained traction over the past decade due in part to the ability to maintain characteristics of the tumor of origin [50,51,52,53]. However, attempts to establish 3D organoid cultures from ccRCC tissue have shown a low rate of success [23]. The basis for many 3D organoid model systems is generally Matrigel, which poorly matches the ECM environment of ccRCC (Figure 2B–E). We postulated that utilization of ccRCC ECM in a 3D culture system would improve both viability of tumor cells and representation of stromal populations from ccRCC tumors. Fibrinogen is an attractive hydrogel substrate due to its tunability, and fibrin gels have been used in wound healing, drug delivery, cell differentiation, and cancer modeling [54]. Fibrinogen is abundantly represented in tumors and can therefore be considered a component of the tumor microenvironment, making it a particularly attractive candidate matrix for in vitro modeling. Differential analysis of tumor versus normal mass spectrometry data from this study showed increases in fibrinogen components FIBA, FIBB, and FIBG in tumors relative to healthy cortex (Table S1), making up a significant percentage of total protein identified in ccRCC tumor samples (FIBA = 4.00%, FIBB = 4.83%, FIBG = 6.79%). Additionally, analysis of the Renal Cancer subset of The Cancer Genome Atlas (TCGA) database identifies fibrinogen genes as highly upregulated in kidney cancer tissue (fibrinogen-High > 60% of samples), indicating that it is locally expressed and not simply deposited by the circulation (Figure S7). From these observations we conclude that fibrinogen is a native ccRCC tumor component that can be used as a versatile matrix to mimic the tumor ECM environment by mixing with ECM components identified in this study.

**Figure 5:**
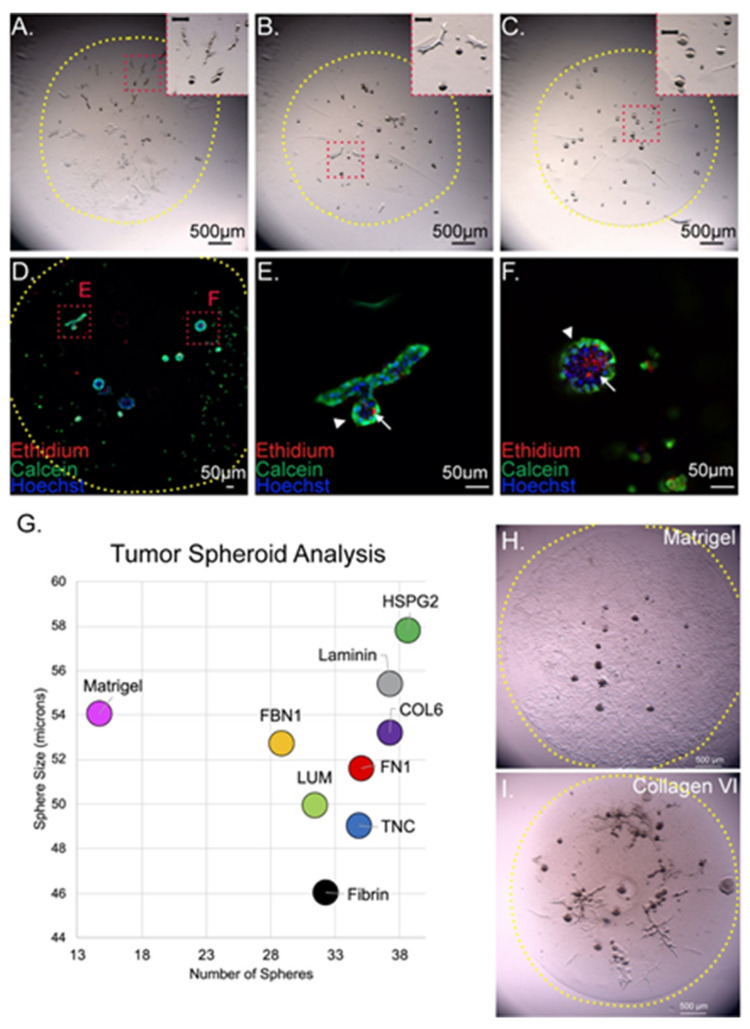
Three-dimensional culture of 786-O utilizing tumor-specific ECM. (**A**–**C**) Modification of fibrinogen concentrations, 2 µg/mL, 4 µg/mL, and 10 µg/mL changes morphology of cell structures formed by 786-O cells. Yellow dotted line outlines region containing 3D dome. Red dotted line indicates region of magnified inset. (**D**–**F**) Live–dead analysis of cell structures found in fibrin domes with 4 mg/mL fibrinogen shows viable elongated structures (**E**) as well as ones with necrotic cores (**F**). Arrowhead indicates viable cells. Arrow indicates dead cells. Blue = Hoechst33342, green = calcein-AM, red = ethidium homodimer 2. (**G**) Analysis of spheroids in Matrigel or fibrin with or without added ccRCC ECM proteins showing changes in number of spheroids and size. (**H**,**I**) Example of 786-O growth in Matrigel versus fibrin mixed with collagen VI (additional single ECM component blends with fibrin are shown in Figure S6). Yellow dotted line outlines region containing 3D dome.

Fibrin 3D cultures were established by mixing single cell suspensions with fibrinogen and thrombin and spotting onto glass coverslips. The surface tension of the glass maintains a dome of cell-fibrinogen mixture that gelates at 37 °C. Dome volumes up to 20 µL gelated within 10 min and were subsequently submerged in medium for the duration of the culture period. Growth of tumor cells in domes was monitored by microscopy throughout the culture period. Fibrinogen concentration determines viscosity of the hydrogel, which determines growth properties of tumor cells. Spheroidal and branching tumor cell aggregates have been identified in previous studies of 3D tumor cell growth. While the spheroidal conformation has been associated with growth of colonies from tumor stem cells [55,56], branching growth patterns have been associated with invasive behaviors of tumors [57,58]. To define a concentration for use in our studies, we analyzed 3D growth patterns of 786-O ccRCC cells in fibrin domes at three concentrations of fibrinogen: 2 mg/mL, 4 mg/mL, and 10 mg/mL. Cells were grown in Matrigel domes as a comparator. One hundred 786-O cells were seeded per 5 µL dome and subsequently cultured for 2 weeks to ensure that the fibrin remained polymerized over an extended culture period (Figure 5A–C). Fibrin dome integrity and tumor cell growth were observed in all conditions, but variations in fibrinogen concentration drastically changed 3D growth patterns. At 2 mg/mL, cells grew predominantly in branching structures (Figure 5A), while a mix of spheroid and branching structures was seen at 4 mg/mL (Figure 5B). At 10 mg/mL, cells grew in spheroids (Figure 5C). Both branched and spheroidal structures contained viable cells (Figure 5D–F). Necrotic centers were observed in spheres larger than 40 microns in diameter (Figure 5F). We reasoned that an intermediate concentration would be best for maintaining heterogeneity in patient-derived samples since it promoted both growth patterns. To verify this, we studied growth patterns of three primary tumor cell lines in fibrin dome cultures at 4 mg/mL fibrinogen concentration and confirmed that we can generate viable multicellular structures with a variety of morphologies (Figure S8). We next asked if we could incorporate ccRCC ECM components into the 3D culture system and if this would enable analysis of 3D cell growth. 786-O cells were grown in Matrigel or 4 mg/mL fibrin domes containing 4 µg/mL ECM proteins (Figure 5G,H and Figure S9). Nine days after seeding, cultures were analyzed for the presence of multicellular spheroid structures (Figure 5I). While larger spheroids were formed in Matrigel, the total number of structures was small, and many cells spread in a monolayer on the glass instead of growing in the 3D gel (Figure 5H). In comparison, cells grown in fibrin generated more multicellular structures (Figure 5I and Figure S9). The incorporation of ECM components into fibrin significantly increased the size of structures (Figure 5G). Interestingly, the inclusion of laminin, a primary component of Matrigel, to fibrin increased spheroid size when compared to fibrin alone while also maintaining a greater number of clusters when compared to Matrigel (Figure 5G). This suggests that other components of Matrigel limit abundance of multicellular structures. In conclusion, we show that fibrin is an appropriate carrier for ccRCC ECM components, can be used to culture both immortalized and primary cell lines, and can be a useful tool for studying the effects that the ECM has on ccRCC growth.

### 3.7. D ccRCC ECM Model Maintains ccRCC Fibroblast Representation

**Figure 6:**
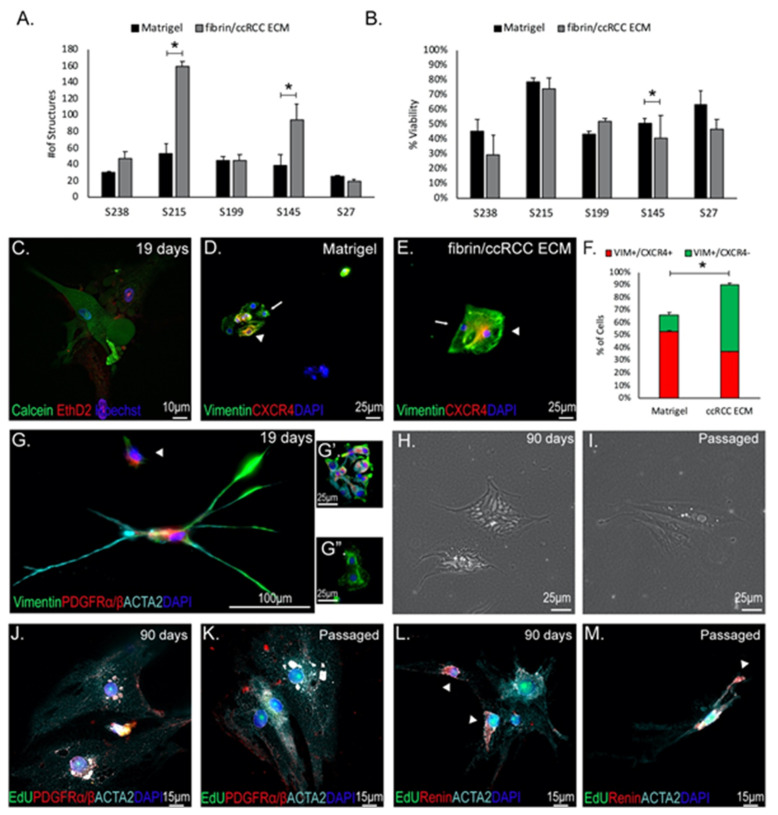
Fibrin/ccRCC ECM 3D culture maintains viable ccRCC fibroblasts. (**A**) Three-dimensional cultures of primary tumor digests culture either in Matrigel or fibrin/ccRCC ECM domes. * *p* ≤ 0.05. (**B**) Viability analysis of structures based on calcein-AM/ethidium homodimer 2 staining. * *p* ≤ 0.05. (**C**) Representative example of viable multicellular structures from patient-derived ccRCC tumor digests cultured in fibrin/ccRCC ECM domes. Green = calcein-AM, red = ethidium homodimer 2, blue = Hoechst33342 (**D**,**E**) Presence of VIM+/CXCR4+ in both Matrigel and ccRCC ECM cultured ccRCC primary digests. Arrowhead indicates CXCR4+ cells and arrow indicates CXCR4- cells. Green = vimentin, red = CXCR4, blue = DAPI. (**F**) Quantification of VIM+ cells in Matrigel versus fibrin/ccRCC ECM shows significantly more positive cells in cultures from fibrin/ccRCC ECM compared to Matrigel. However, significantly more VIM+/CXCR4+ are found in Matrigel compared to fibrin/ccRCC ECM * *p* ≤ 0.05 (**G**,**G’**) Multicellular structures show expression of fibroblast markers PDGFRα/β (red) and vimentin (green), as well as presumed ccRCC CAF marker ACTA2 (cyan). Blue = DAPI. Arrowhead indicates presence of PDGFRα/β+/ACTA2- cells. (**G”**) Some VIM+ structures do not express PDGFRα/β. Green = vimentin, red = PDGFRα/β, blue = DAPI. (**H**–**K**) Fibrin/ccRCC ECM cultures were maintained for 90 days (**H**) or passaged 1:4 into fibrin/ccRCC ECM (**I**), at which point EdU was incorporated for 48 h. Cultures were immunostained (**J**,**K**) for EdU (green), PDGFRα/β (red), and ACTA2 (cyan). Blue = DAPI. (**L**,**M**) Cultures were immunostained additionally for renin (red). Arrowheads indicate renin+ cells. Green = EdU, cyan = ACTA2, blue = DAPI. White staining is overlap between red and cyan. Arrowheads indicate renin+ cells.

Having established both that ccRCC ECM supports attachment of diverse cell types isolated from primary tumors, and that these factors can be mixed into a fibrin hydrogel matrix that supports 3D tumor cell growth, we wanted to know how this tumor-mimicking 3D environment affects growth of primary tumor isolates compared to the current standard Matrigel. We digested five different patient tumor samples into single cells using previously described methods [23]. We then seeded 750–1000 viable cells into replicate 3D domes of either Matrigel or fibrin/ccRCC ECM. Three-dimensional cultures were maintained for 19 days, at which point we analyzed the number of multicellular structures and cell viability using Calcein-AM and Ethidium Homodimer-2 (Figure S10). As expected, significant variation was observed between patient samples. For all samples, multicellular structures were seen in both fibrin/ccRCC ECM and Matrigel domes. However, for samples S215 and S145, significantly more 3D structures were seen in ccRCC ECM compared with Matrigel (Figure 6A). Approximately half of the structures seen in all samples were composed of viable cells in both fibrin/ccRCC ECM and Matrigel, and S145 displayed significantly more viable cells in Matrigel (Figure 6B,C). Interestingly, the lowest viability was seen in the samples with most abundant 3D structures. To understand if viability could be improved by culture in medium tailored to tumor organoids, we compared viability of cells from sample S215 in our growth medium versus CHM growth medium developed specifically for tumor organoid culture [23]. Tumor organoid medium significantly reduced cell viability in Matrigel, fibrin alone, and in fibrin/ccRCC ECM domes (Figure S11), and further studies were conducted using standard growth medium. From these studies we conclude that fibrin/ccRCC ECM can be used to propagate 3D structures from primary tumor isolates with a similar efficiency to Matrigel.

To understand which cell types are represented in 3D cultures from primary tumor isolates grown using ccRCC ECM versus Matrigel, we immunostained them for molecular markers on day 19 of culture. Vimentin and CXCR4 are widely used to identify cell types within tumor cultures; co-staining differentiates vimentin-positive CXCR4-negative (VIM^+^/CXCR4^−^) presumptive fibroblasts from vimentin and CXCR4 co-expressing (VIM^+^/CXCR4^+^) presumptive cancer stem cells that have previously been reported to grow out in 3D Matrigel cultures [59,60]. As expected, clusters of VIM^+^ tumor cells were prevalent throughout cultures established in both Matrigel domes (Figure 6D and Figure S12) and fibrin/ccRCC ECM cultures (Figure 6E and Figure S12). Quantification revealed that 66% of cells in Matrigel were VIM^+^ whereas over 90% of cells in fibrin/ccRCC ECM were VIM^+.^ (Figure 6F). Comparison of CXCR4 expression within the VIM^+^ cell populations showed that CXCR4 was expressed in over 80% of cells in Matrigel domes, as expected (Figure 6F). However, only in 41% of VIM^+^ cells were CXCR4^+^ in fibrin/ccRCC ECM (Figure 6F). From this we conclude that approximately 55% of cells in 3D Matrigel cultures are VIM^+^/CXCR4^+^ presumptive tumor cells, and approximately 10% are VIM^+^/CXCR4^−^ presumptive fibroblasts. In fibrin/ccRCC ECM cultures approximately 37% of cells are VIM^+^/CXCR4^+^ presumptive tumor cells, and 53% are VIM^+^/CXCR4^−^ presumptive fibroblasts. Our previous studies and published reports have shown that fibroblasts fail to thrive in Matrigel, and we were therefore interested in understanding the identities and growth properties of the VIM^+^/CXCR4^−^ presumptive primary fibroblasts in Matrigel versus fibrin/ccRCC ECM cultures.

To compare fibroblast representation, cultures were stained for the fibroblast marker PDGFRα/β and, as previously observed in this study, few PDGFRα/β^+^ fibroblasts were found in Matrigel cultures (less than 3% of cells in the culture) (Figure 1F and Figure S13). However, abundant clusters of fibroblasts were observed in the fibrin/ccRCC ECM cultures (more than 33% of cells in the culture) (Figure 6G,G’ and Figure S13). Interestingly, we also found expression of ACTA2 in many PDGFRα/β^+^ clusters in fibrin/ccRCC ECM cultures (Figure 6G, arrowhead; 16.5% ACTA2^+^ in the culture). In contrast to Matrigel, the fibrin/ccRCC ECM 3D culture method maintains robust representation of ccRCC fibroblast populations from primary tumor isolates.

To understand if ccRCC fibroblasts can be maintained in fibrin/ccRCC ECM 3D culture, we took two separate approaches. First, we extended the culture period to 90 days to understand if fibroblasts remained viable over longer periods in this culture system (Figure 6H). Second, we subcultured ccRCC ECM domes into fibrin/ccRCC ECM domes to determine if it was possible to propagate fibroblasts (Figure 6I). EdU was incorporated into the culture medium to assay for proliferation in both experiments (Figure 6J–M). Following long-term culture, 28.5% of cells were PDGFRα/β^+^/ACTA2^+^ and showed EdU incorporation (Figure 6J and Figure S14), revealing very little decline in fibroblast number from the 33% proportion quantified at 19 days and suggesting that they continue to proliferate to maintain their proportion of the total culture. Following subculture, 63.6% of cells in the second passage were PDGFRα/β^+^/ACTA2^+^ fibroblasts with EdU incorporation (Figure 6K), showing that these cells can be propagated in these conditions without losing marker expression. The proportion of fibroblasts isolated directly from tumors is approximately 7% (Figure 1C), indicating that these cells are advantaged in our culture conditions. Furthermore, their proportion of the total culture can be expanded by passaging.

Although the renin expressing subset of fibroblasts is small in patient ccRCCs, transcriptome analysis indicates that it may be a significant contributor of ECM proteins (Figure 3B). To understand if this cell type is maintained following extended culture or subculture, we co-stained samples for ACTA2 and renin. Following 90 days of culture, analysis of ACTA2^+^ cells show that 29.2% are renin^+^, and following subculture, 27.7% are renin^+^ (Figure 6L,M and Figure S14). In both cases, ACTA2^+^/renin^+^ were labeled with EdU indicating that they are proliferative (90-day culture = 21.42% EdU^+^; subculture = 31.8% EdU^+^). Thus, the unique and poorly understood ccRCC CAF population is maintained and propagated in fibrin/ccRCC ECM with maintenance of the distinct tumor fibroblast repertoire.

## 4. Discussion

Neoplastic transformation of an epithelial cell initiates tumor formation, but the interaction of the transformed cell with non-transformed cells in its environment controls tumor formation. Numerous studies have shown that the reciprocal interactions between transformed cells and their untransformed environments are highly complex and should be considered an aberrant form of organogenesis [61]. Genetic evidence supports inactivation of the oxygen sensor VHL as the initiating event in ccRCC, with the resulting pseudo-hypoxia promoting a persistent state of angiogenic recruitment in the transformed cell [62]. The axis of communication between tumor cells and endothelium has been a major research focus that has yielded effective therapies [63]. How tumor cells interact with surrounding cells to control the immunological environment and escape lymphocyte attack has also become a major research question given the success of immunotherapies [64]. Studies in tumor biology and developmental biology support an essential role for fibroblasts in regulating both angiogenesis and the local immune environment [65,66,67]. Our study reveals the complex requirements required for growth of these cells and provides a strategy to culture them that may be used to answer basic questions regarding their influence on tumor cell growth and immunomodulation.

ECM governs cell behaviors through complex mechanisms including acting as a sink for growth factors and controlling tissue stiffness and elasticity. For this series of experiments, the cell attachment properties of ECM are of particular interest. Cells associate with ECM molecules such as collagens, laminin, and fibronectin through receptors at the cell surface that include integrins, the laminin receptor, syndecans, and dystroglycan. These have different affinities for distinct ECM components and are generally redundantly expressed, forming a cell-specific ECM-binding signature. Interactions between these surface proteins and ECM components are the basis of physical association of cells with the tissue scaffold. For this reason, it is important to accurately reproduce the ECM protein repertoire in culture so that the combination of ECM molecules required for the attachment of diverse cell types liberated from dissociated tumors are represented. Our analysis defined specific differences in ECM-binding affinities between the predominant cell types found in tumors. However, we showed that by generating a blend of the most abundant tumor ECM components with proportions reflecting those in the ccRCC tumors we could promote binding of all cell types. Capturing the cells within the culture system is an essential first step towards establishing long-term cultures of patient tumors, and further investigations of metabolite composition and oxygenation of medium will guide efforts to grow cells in 3D matrix to a density similar to that seen in tumors.

In addition to their role in cellular adhesion, ECM-binding cell surface receptors control cytoskeletal contacts with the surroundings. Our study identified an interesting contrast between the ECM of healthy neighboring kidney cortex and tumors. In the healthy kidney, epithelial cells sit on basement membranes and stromal cells are embedded within the adjacent interstitial ECM which functions as a scaffold for the organ. The compositions of basement membranes and interstitial ECM are highly distinct, but ccRCC appears to consist of a mixture of components, with interstitial ECM components most highly represented. Thus, tumor cells and stromal cells are embedded in a single ccRCC ECM and this unique structural arrangement is anticipated to have profound effects on cell behaviors. Culture systems based on outgrowth of cells from tumor fragments including native tumor ECM have been successfully used to grow mixed cultures of tumor and stromal cells [68]. We speculate that the mismatch in ECM profile between Matrigel, which mimics basement membrane, and fibrin/ccRCC ECM, which largely contains interstitial ECM components, may be the reason that the few fibroblasts that do adhere to Matrigel are rapidly lost in culture. A previous report comparing the growth of patient-derived ccRCC tumor cells in Matrigel versus a polysaccharide scaffold found little growth of fibroblasts in Matrigel and suggested that polysaccharide biomaterials would be preferable for outgrowth of non-tumor cells [69]. The rounded structure of fibroblasts in Matrigel suggests aberrant cytoskeletal arrangement consistent with a lack of ECM interaction. Fibroblasts in ccRCC ECM display characteristic morphology and are maintained in culture.

A defining feature of the ccRCC ECM composition characterized in this work is its qualitative similarity to healthy kidney cortex ECM. Within the detection limits of our analysis, neoplastic transformation does not lead to de novo expression of matrix molecules but rather alters the relative abundances of components. In contrast to healthy cortex, ccRCC ECM is highly enriched in collagen VI, fibronectin, tenascin C, TGFBI, and periostin. The role of each of these components has been studied in tumor cell biology. Collagen VI is abundantly expressed in tumors from several organs including breast [70], colon, and lung [71]. It promotes survival of tumor cells [72] and fibroblasts [73] and has been shown to stimulate tumor angiogenesis [74]. Expression of collagen VI in xenografted ccRCC cells results in increased tumor size [75]. Fibronectin promotes tumor growth through activation of PI3K/AKT signaling in tumor cells [76,77]. Tenascin C closely resembles fibronectin and shares receptor-binding properties, promoting proliferation [78] and migration [79] of tumor cells. Similarly, TGFBI promotes proliferation and migration of cancer cells [80]. Periostin promotes tumor cell growth through increased survival [81] and expression in xenografted ccRCC cells promotes tumor size [82]. All of these ECM proteins may activate multiple signal transduction pathways through their cell surface receptor interactions, including PI3K, TGFβ, ERK, and STAT.

In many tumors, the alteration in ECM content results in a more rigid matrix that promotes malignancy [83], but in ccRCC we have found that this is not the case: modulus testing of tumors versus adjacent healthy cortex revealed a lower modulus in tumors [84]. This suggests that the ECM profile of ccRCCs defined in our analysis reduces tissue rigidity in comparison with the healthy surrounding tissue, perhaps contributing to the indolent progression of ccRCC tumors, which genetic analysis has revealed generally develop over decades [85].

Our reanalysis of published single cell data from ccRCCs indicates that tumor fibroblasts serve as a major source of ECM. This is certainly in line with observations from tumors that develop in other organ systems [86], where myofibroblast-derived ECM has been identified as a major determinant of the tumor microenvironment. Interestingly, we found that PDGFRα/β-expressing fibroblasts identified in the Young et al. single-cell dataset [33] segregated into two distinct clusters; the less abundant subset of these had high expression of ACTA2 (smooth muscle actin), identifying them as the putative myofibroblast population. In agreement with published reports on myofibroblast production of ECM, this subset of cells displayed strong expression of ECM components identified by mass spectrometry; collagen VI, HSPG2/perlecan, fibronectin, lumican, laminin, and collagen 12. Thus, we propose that these cells play a major role in forming the ECM environment of ccRCC. Immunostaining reveals that ACTA2-expressing cells surround clusters of clear cells in ccRCC tissue, indicating that they deposit their matrix within the network of stroma that contains vessels and immune cells. Our study indicates that these ccRCC myofibroblasts express renin, an angiotensinogen protease that promotes vasoconstriction through the renin-angiotensin (RAS) cascade. The presence of a renin expressing cell in ccRCC stroma has previously been reported [49], and subsequent work has identified it as a cancer stem cell [87]. However, based on our findings we propose that it is a myofibroblast subpopulation of CAFs in ccRCC. In the healthy kidney, renin is expressed in a specialized subpopulation of cells within vessel walls [88] that is essential for maintaining blood pressure by secreting renin in response to neural and chemical cues [89]. Clinical observations support a role for the RAS system in ccRCC progression, with patients treated with angiotensin inhibitors displaying improved survival in metastatic ccRCC [90]. A recent study showed that RAS inhibition prevents ccRCC tumor colony formation, indicating direct effects of this pathway on tumor tissue [91]. Contribution of the ccRCC myofibroblast to activation of the RAS system in ccRCC is therefore an intriguing possibility. Our demonstration that the renin-expressing myofibroblast can be propagated in culture provides a novel and unique tool to investigate specific roles of the ccRCC CAF in tumor biology.

## 5. Conclusions

Proteomic analysis identified drastic changes in the ECM environment of ccRCC compared with healthy neighboring kidney cortex tissue, including structural changes such as loss of tubule basal lamina and alterations in the composition of ECM. scRNA-seq analysis identified a subset of CAFs with a strong transcriptomic profile for ECM expression, identifying them as the source of increased matrix deposition. We were unable to culture these ACTA2 and renin expressing CAFs using conventional cell culture techniques, including monolayer and Matrigel domes. To isolate and propagate these cells we devised a ccRCC specific ECM combination (ccRCC ECM) based on the proteomic profiles of patient ccRCCs. Cell types representative of the tumor showed efficient attachment to this substrate and incorporation of this ccRCC-characteristic ECM mix into fibrin gels enabled creation of a tunable 3D ccRCC environment (fibrin/ccRCC ECM). Patient tumor-derive primary ccRCC cell isolates grew out in this 3D tumor culture, and molecular marker analysis revealed representation of both tumor cells and fibroblasts. The fibroblast repertoire found in tumors was preserved with subsets of cells showing high expression of ACTA2 and renin. In conclusion, we found that the use of ccRCC specific ECM components in a fibrin-based 3D culture system allows for robust culture of ccRCC fibroblasts for use in studying tumor-CAF-ECM interactions, as well as further exploration of different fibroblast subtypes within ccRCC tumors.

## Figures and Tables

**Table 1:** Tissue samples used in this study. Stage and grade determined by pathologist using the TNM and Fuhrman systems, respectively. p = primary T = Tumor N = lymph nodes (x = not determine, 0 = none detected, 1 = spread) M = metastasis (x = not determined, 0 = none detected, 1 = spread).

ID	Diagnosis	Stage	Grade	Sex	Race	Age	Applications
Tp17-S560	ccRCC	pT3 Nx Mx	3	M	White	72	Mass Spec
Tp18-S435	ccRCC	pT3a NX	4	M	White	50–59	Mass Spec
R19-6098	ccRCC	pT3 pN0	2	N/A	White	29	Mass Spec
Tp19-S80	ccRCC	pT3a N0 Mx	4	M	White	69	Mass Spec
Tp19-S79	ccRCC	pT3a Nx	4	F	White	68	Mass Spec
Tp18-S109	ccRCC	pT3a	2	M	White	60–69	Mas Spec, Primary Culture
Tp18-S114	ccRCC	pT3a	3	M	White	80–89	Mass Spec, Primary Culture
Tp18-S108	ccRCC	pT1a Nx Mx	2	F	White	60–69	Primary Culture
R18-6256	ccRCC	pT1a pNC	1	N/A	N/A	61	Flow Cytometry
R19-6200	ccRCC	pT1b Nx	2	M	White	61	Flow Cytometry
Tp18-S601	ccRCC	pT1b Nx	2	M	White	61	Flow Cytometry
R18-1453	ccRCC	pT2a pNx	3	F	White	83	Flow Cytometry
Tp21-S145	ccRCC	pT3a Nx Mx	2	F	White	76	3D Culture
Tp20-S199	ccRCC	pT3a Nx	2	M	N/A	74	3D Culture
Tp21-S27	ccRCC (Syndromic VHL)	pT1a Nx Mx	4	M	White	68	3D Culture
Tp20-S215	ccRCC	pT4 Nx M1	4	M	White	52	3D Culture
Tp20-S238	ccRCC	PT3 + Nx M1	4	M	White	71	3D Culture

## Data Availability

SWATH and ion library raw data will be located at the PeptideAtlas repository, and accessible via the web. PeptideAtlas is a part of the ProteomeXchange Consortium. scRNA-seq data from Young et al. [33] and can be downloaded from the European Genome-phenome Archive (EGA) under study IDs EGAS00001002171, EGAS00001002486, EGAS00001002325 and EGAS00001002553. ProteinAtlas staining data are publicly available at https://www.proteinatlas.org (accessed on 6 January 21). Data from The Cancer Genome Atlas are publicly available from the TCGA repository, https://portal.gdc.cancer.gov/projects/TCGA (accessed on 6 January 21).

## Supplementary Materials

The following are available online at https://www.mdpi.com/article/10.3390/cancers13235873/s1, Supplemental Methods; Figure S1: Flow-cytometry-based cell type marker analysis; Figure S2: Loss of PDGFRα/β after passaging primary ccRCC cultures; Figure S3: Pathway analyses of ccRCC mass spectrometry dataset; Figure S4: Cell attachment on ECM; Figure S5: ccRCC cell marker analysis of primary cell lines; Figure S6: EdU analysis of primary tumor isolates; Figure S7: Analysis of fibrinogen transcripts from TCGA; Figure S8: Primary RCC cultures in fibrin domes; Figure S9: Incorporation of ECM into fibrin domes; Figure S10: ccRCC tumor isolate culture in Matrigel vs. fibrin/ccRCC ECM; Figure S11: Media formulation influences cell viability of 3D cultures; Figure S12: Further characterization of 3D cultures; Figure S13: Immunostaining analysis of fibrin/ccRCC ECM cultures for fibroblast markers; Figure S14: Additional analysis of fibroblast markers after 90 days of culture; Figure S15: Protein localization validation; Table S1: Mass Spectrometry Matrisome Analysis; Table S2: Gene Ontology (GO) Analysis; Table S3: Gene Set Enrichment Analysis (GSEA); Table S4: Database for Annotation, Visualization and Integrated Discovery (DAVID) Analysis; Table S5: RCC scRNA-seq Analysis; Table S6: Fibroblast clusters analysis.
