# Supplementary material for: The Extracellular Matrix Environment of Clear Cell Renal Cell Carcinoma Determines Cancer Associated Fibroblast Growth
Source: Cancers (Basel). 2021 Nov 23;13(23):5873. doi: 10.3390/cancers13235873 (PMC8657052; doi:10.3390/cancers13235873)
Supplement: Supplementary file 1 [file cancers-13-05873-s001.zip › cancers-1428187-Supplementary Methods and Figures.pdf]

cancers MDPI

Supplementary Material: The Extracellular Matrix Environment of Clear Cell Renal Cell Carcinoma Determines Cancer Associated Fibroblast Growth

Kyle H. Bond, Takuto Chiba, Kieran P.H. Wynne, Calvin P.H. Vary, Sunder Sims-Lucas, Jeannine M. Coburn and Leif Oxburgh

Supplemental Methods

S1.1 Mass Spectrometry

S1.1.1 Trypsin Digestion/Peptide Normalization

All chemicals were LCMS or HPLC grade or better. Approximately 50 mg of each tissue sample was re-suspended in 400 ul of 8 M Urea/50 mM Tris-HCL pH8 with phosphatase and protease inhibitors (Roche) then sonicated (Branson Sonifier 250, Branson Ultrasonics) for 3x10 seconds) and rested on ice. 50 µl of each lysate was reduced for 30 minutes with 8 mM dithiothreitol (dtt) and alkylated for 15 minutes with 20 mM iodoacetamide at 30°C. The 8 M urea solution was diluted with 300 µl of 50 mM Tris-HCL pH8 and samples were digested overnight with 20 µg sequencing grade trypsin (Promega, Trypsin Protease, MS Grade) (Optimal Ratio of Trypsin to Substrate 1:20 – 1:100). From each sample 100 µl of the trypsin digested lysate was cleaned and normalized to approx. 30 µg using Pierce C18 Spin Columns. (ThermoFisher Scientific). In parallel the remaining lysate from each sample (approx. 250 µl) was cleaned using Glygen TopTip C18 spin columns pooled and dried using a vacuum centrifuge ready for offline fractionation.

S1.1.2 Basic Reverse Phase (bRP) C18 Chromatography

The dried pooled peptide fractions were re-suspended in 100 µl of 98% hplc grade water/1.9% HPLC grade acetonitrile/0.1% ammonium hydroxide. An Agilent1100 HPLC system was used to resolve samples over a Jupiter 5µ C18 300A 250 mm x 4.6mm chromatography column (Phenomenex) with a 22 minutes increasing acetonitrile gradient. 20 fractions were collected and merged into 10, dried using vacuum centrifugation ready for mass spectrometry.

S1.1.3 Mass Spectrometry

Trypsin digested samples were run on a Sciex TripleTOF 5600 mass spectrometer connected to a Dionex Ultimate 3000 (RSLCnano) chromatography system, as described previously. Tryptic peptides were re-suspended in 0.1% formic acid. Each sample was loaded onto a reverse phase C18 nano column (40 cm length, 75 µm ID) packed with Reprocil Pur C18,1.9 µm (Dr Maisch) and resolved by an increasing acetonitrile gradient over 120 minutes at a flow rate of 220 nL/minute. The mass spectrometer was operated using data dependent acquisition (DDA) to create a library. Sequential window acquisition of all theoretical spectra (SWATH) was implemented for relative quantitation. For both modes of operation (DDA and SWATH) the mass spectrometer used an ion spray voltage floating (ISVF) of 2400 V, curtain gas (CUR) 25 PSI, interface heater temperature (IHT) 150 °C, ion source gas 1 of 6 PSI and a declustering potential (DP) 100 V

All data acquired in data dependent acquisition (DDA) mode used a high resolution MS scan from 350-1500 m/z to select the 50 most intense ions prior to MS/MS analysis using CID (Collision Induced Dissociation). Other parameters include; charge states 2-5, exclusion time 30 seconds accumulation time of 250ms for TOF MS and 50ms for TOF MS^2, cycle time 2.8 seconds.

Unique SWATH parameters included 1 TOF MS scan with an accumulation time of 96 ms followed by 100 variable scan windows from 350 to 1500 m/z, accumulation time of

89.9 ms, cycle time 9.1 seconds. Identical chromatography parameters were used for SWATH and IDA analysis.

S1.1.4 Liquid Chromatography

From 0-9 minutes the sample is loaded (using the loading pump) on to a C18 trap column (Thermo Scientific) at a flow rate of 3 ul/minutes. At 9 minutes a switching valve was used to switch the C18 trap column in line with the gradient pump. From 0-1 minutes buffer b increases from 0-1% at a flow rate of 220 nl/minute (using the gradient pump), from 1-104 minutes buffer b increases from 10-35%, from 104-108 minutes buffer b increases from 35-95%, from 108-118 minutes buffer b remains at 95%, from 118-119 minutes buffer b decreases from 95-2%, from 119-120 minutes buffer b remains at 2%. The total length of the gradient is 120 minutes.

Buffer B: 80% acetonitrile, 19.9% water, 0.1% formic acid.

Buffer A: 98% water, 1.9% acetonitrile, 0.1% formic acid.

S1.1.5 Data Analysis

The TripleTOF raw data was searched against a human UniProt database using ProteinPilot software (Version 5.0.2, Sciex) with the Paragon algorithm for the creation of a protein library. Each peptide used for protein identification met specific Protein Pilot parameters i.e. only peptide scores that corresponded to a peptide confidence of greater than 99% were accepted. The database search parameters tryptic digestion, fixed modification of cysteine alkylation (57.02146) with an emphasis on Biological variable modifications were used.

Spectral alignment and targeted data extraction of DIA samples were performed with the SWATH Processing Micro App in PeakView (Version 2.2.0, Sciex) using the reference spectral library generated earlier. The data was imported into MarkerView (Version 1.2.1, Sciex), where data was normalized, groups compared by principal component analysis and mined for significant differences (T-Test)

Raw data was also searched using the search engine Maxquant (release 1.6.4.0) (Jürgen Cox), for peptides cleaved with trypsin. Each peptide used for protein identification met specific Maxquant parameters, i.e. only peptide scores that corresponded to a false discovery rate (FDR) of 0.01 were accepted from the Maxquant database search. The protein intensity of each identified protein was used for relative quantitation.

*MLR normalization

** Unsupervised principal component analysis (PCA)

S1.2 Pathway Analysis

S1.2.1 DAVID Analysis

Processed SWATH data was functionally annotated using DAVID with cutoffs for significant differential expression (p-val < 0.05) and fold change (≥ 1.5-fold change) for both upregulated and downregulated proteins, using the grouped fold change value. Only databases detailing specific cellular signaling pathways or identities were used, including the following: OMIM_DISEASE, COG_ONTOLOGY, UP_KEYWORDS, GOTERM_BP_DIRECT, GOTERM_CC_DIRECT, GOTERM_MF_DIRECT, BIOCARTA, and REACTOME_PATHWAY. Similarity Term Overlap was set to 4, with a threshold of 0.35. Group membership limit was set to 4, with a multiple linkage threshold of 0.50. Only significantly enriched pathways were processed (EASE = 0.05). Upregulated and downregulated enrichment clusters were manually annotated based on identifying keywords found in each, and graphed based on enrichment score. Exported data can be found in Table S1.

S1.2.2 Gene Set Enrichment Analysis (GSEA)

Processed data was analyzed for signaling pathway enrichment using the GSEA with a cutoff for differential expression (p-val < 0.05) and using the raw "real" grouped

intensity values. The GSEA was configured to use the Reactome database for reference. Classic enrichment statistic was used and ranked using Signal2Noise. Sets were collapsed using the Max_probe value, normalized using meandiv, and randomized with no_balance. Data was plotted for visualization using the Cytoscape plugin with a P-value cutoff of 0.005, FDR Q-value Cutoff of 0.01, and Similarity Cutoff using the Jaccard+Overlap Combined value of 0.375. Exported data can be found in Table S1.

S1.2.3 Gene Ontology (GO) Analysis

Processed SWATH data was analyzed for gene ontology enrichment using PANTHERdb with cutoffs for significant differential expression ($p\text{-val} < 0.05$) and fold change (≥ 1.5-fold change), using the grouped fold change value. Exported data can be found in Table S1.

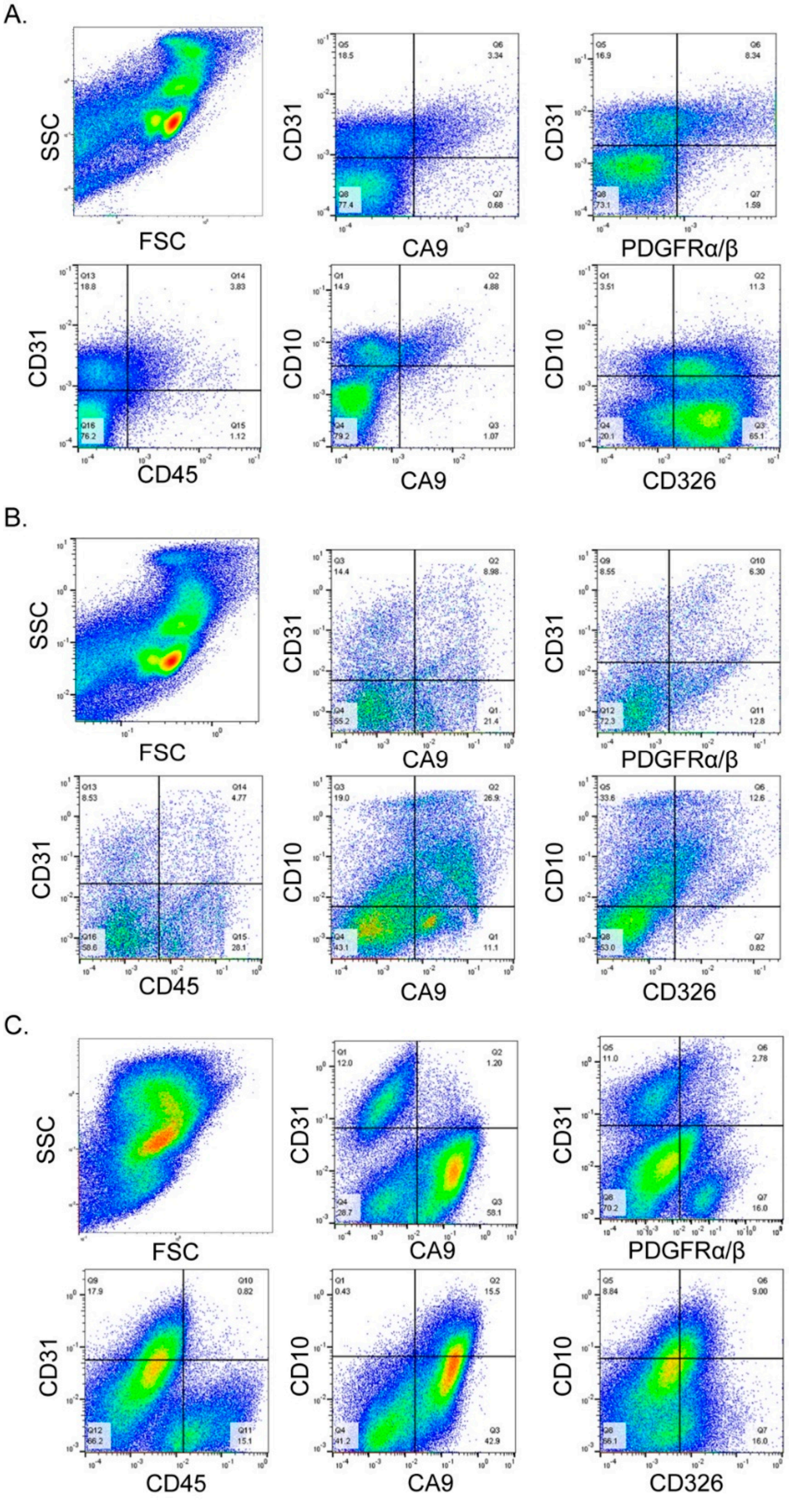

Figure S1. Flow-cytometry-based cell type marker analysis of three tumors. (**A**) Patient R19-6200, (**B**) patient Tp18-S601, and (**C**) patient R18-1453 were analyzed for cell type specific markers using flow cytometry.

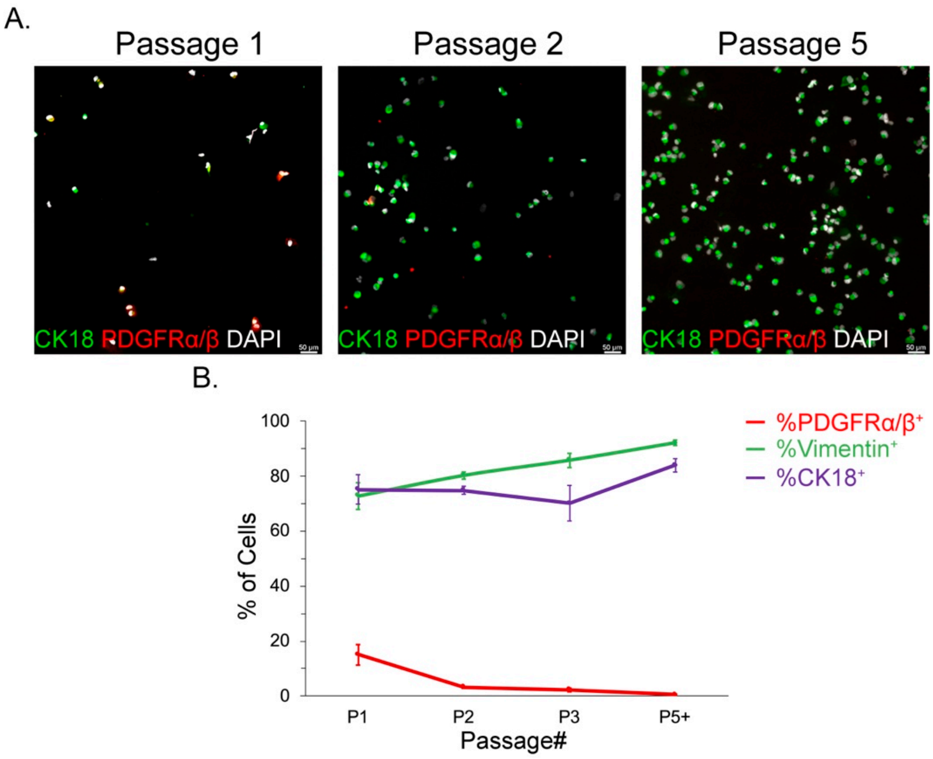

Figure S2. Loss of PDGFRα/β after passaging primary ccRCC cultures. (**A**) Representative images of immunostained patient tumor line Tp18-S108 for tumor cell and fibroblast markers at sequential passages. Green = CK18, red = PDGFRα/β, gray = DAPI. Scale bar = 50 μm. (**B**) Summary analysis of 5 patient derived primary RCC cultures over passages for expression of fibroblast marker PDGFRα/β, tumor marker CK18, and vimentin.

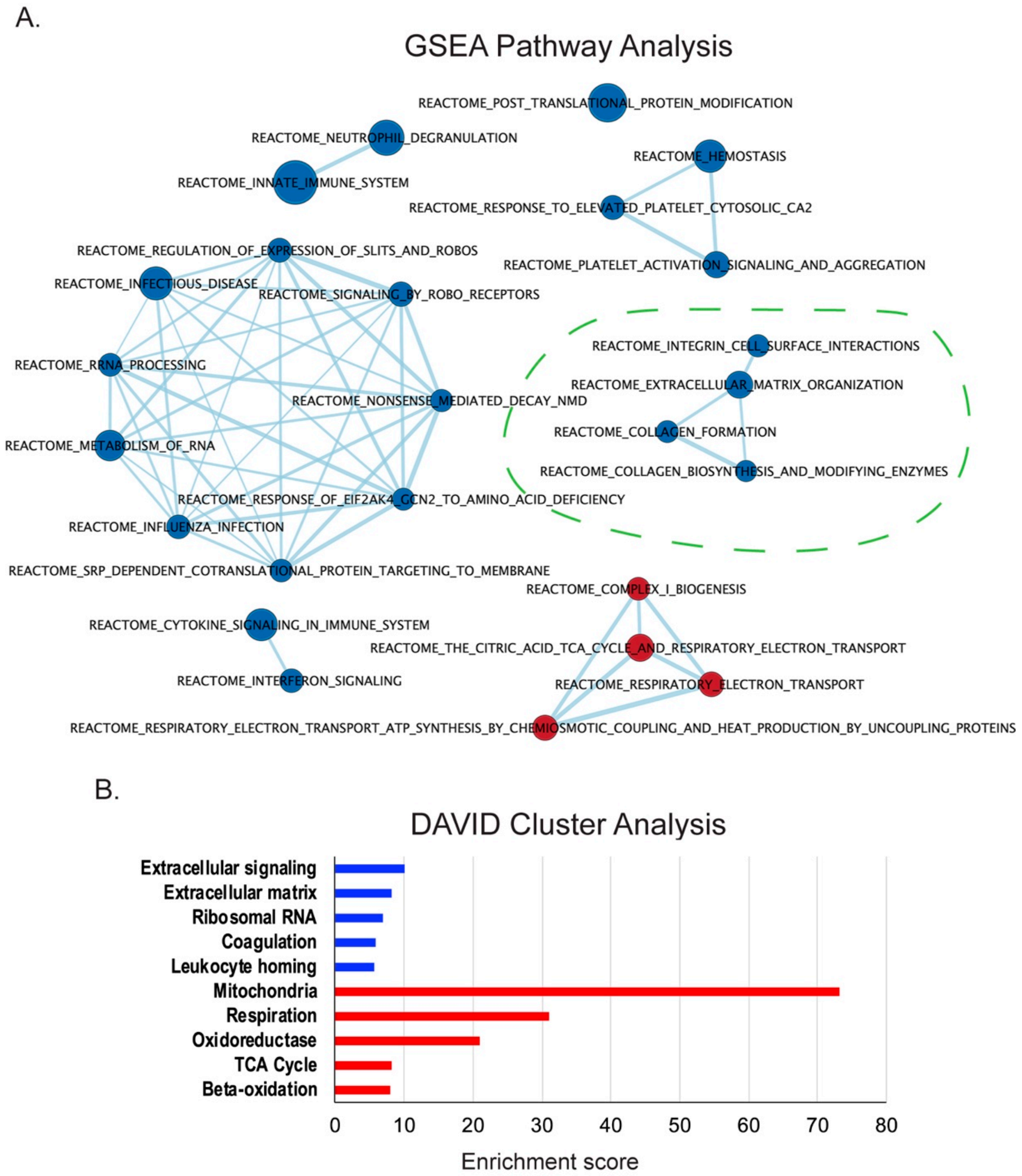

Figure S3. Pathway analyses of ccRCC mass spectrometry dataset. (**A**) Gene Set Enrichment Analysis (GSEA) using the Reactome reference pathway database visualized using Cytoscape. Blue = upregulated in tumors, red = downregulated in tumors. Encircled node indicates increase in ECM related pathways and interactions. (**B**) DAVID enriched analysis of only significantly modified pathways. Red = upregulated, blue = downregulated.

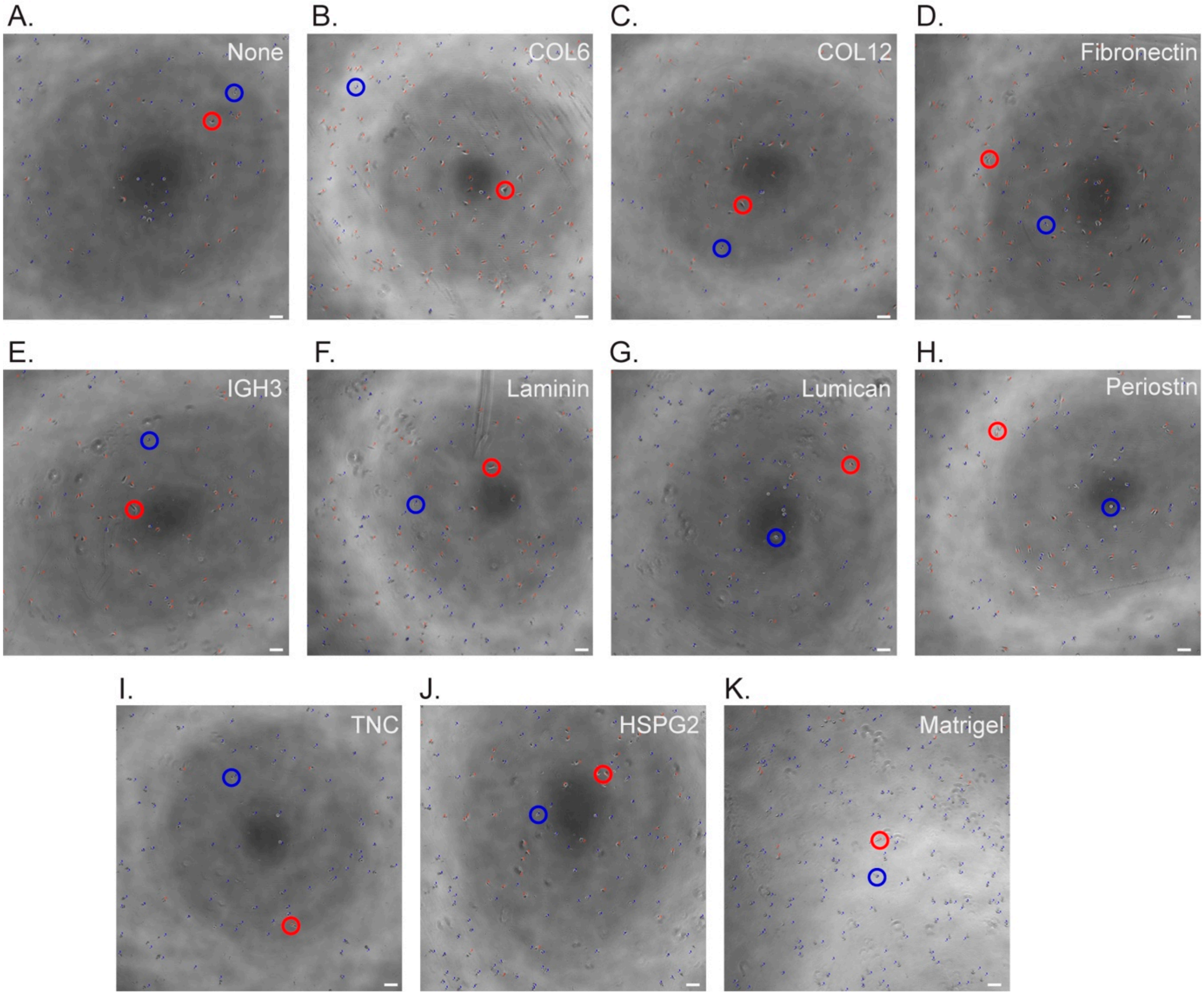

Figure S4. Cell attachment on ECM. (**A**–**K**) Characteristic images of 786-O cell attachment after 2 hours to coated assay plates. Each well was coated with 2 µg/ml of designated recombinant protein. Cell attachment was quantified morphologically (*n* = 3). Blue circle = sample of unattached cell. Red circle= sample of attached cell. Scale bar = 100 µm.

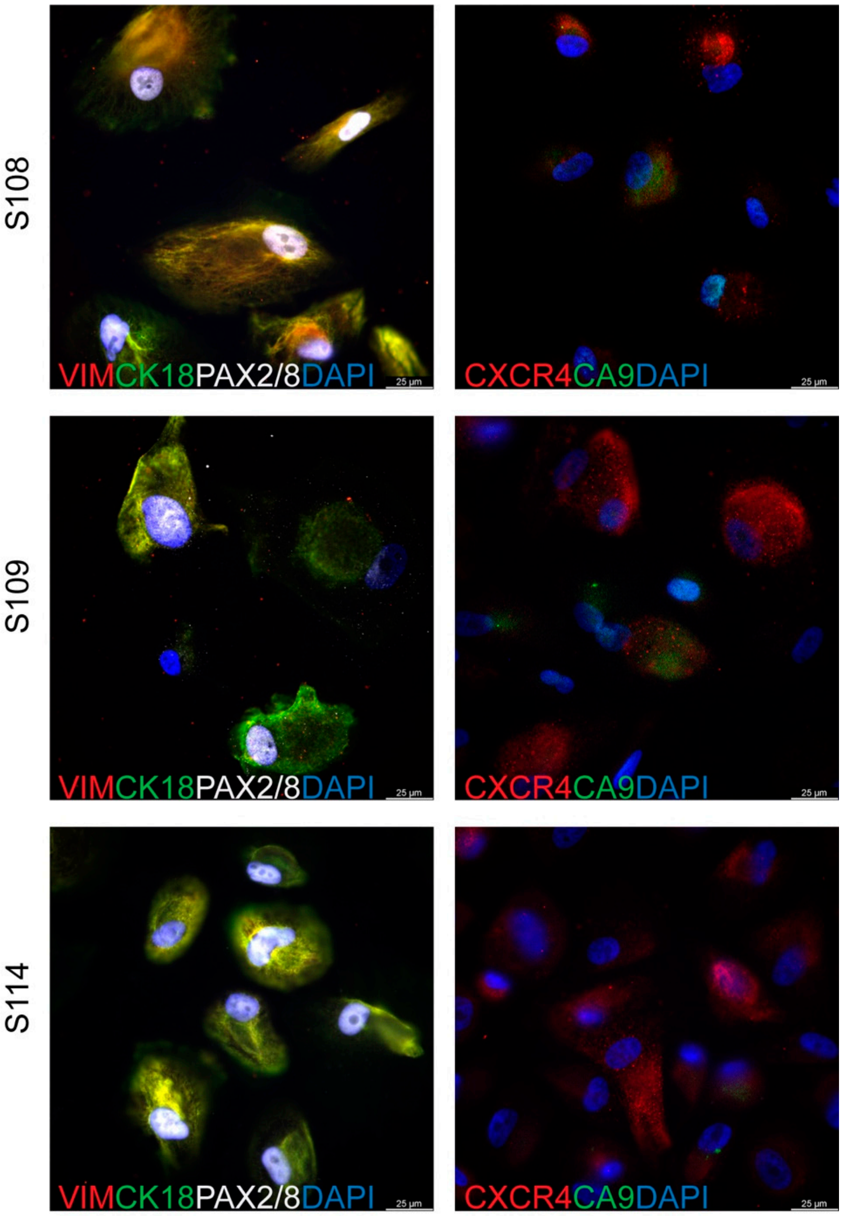

Figure S5. ccRCC cell marker analysis of primary cell lines. Primary ccRCC tumor lines were immunostained for common ccRCC and cancer stem cell markers. Left column; red = vimentin, green = CK18, white = PAX2/8, blue = DAPI. Right column; red = CXCR4, green = CA9, Blue = DAPI. Scale bar = 25 μm.

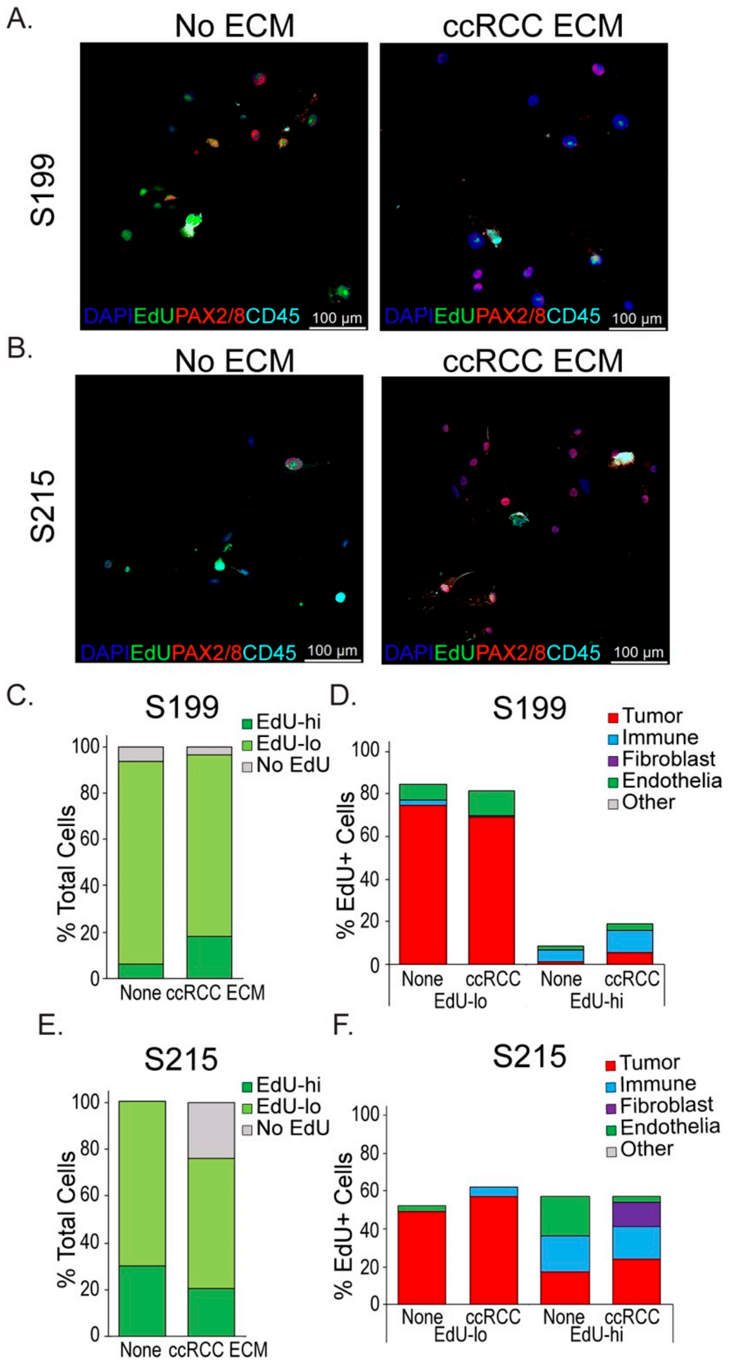

Figure S6. EdU analysis of primary tumor isolates. (**A**–**B**) Representative immunostaining and EdU detection on primary tumor digests cultured on ccRCC ECM coated slides or slides without any coating. Green = EdU, red = PAX2/8, cyan = CD45, blue = DAPI. (**C**–**F**) Analysis of EdU intensity in each cell, further defined by co-expression of other cell-type specific markers.

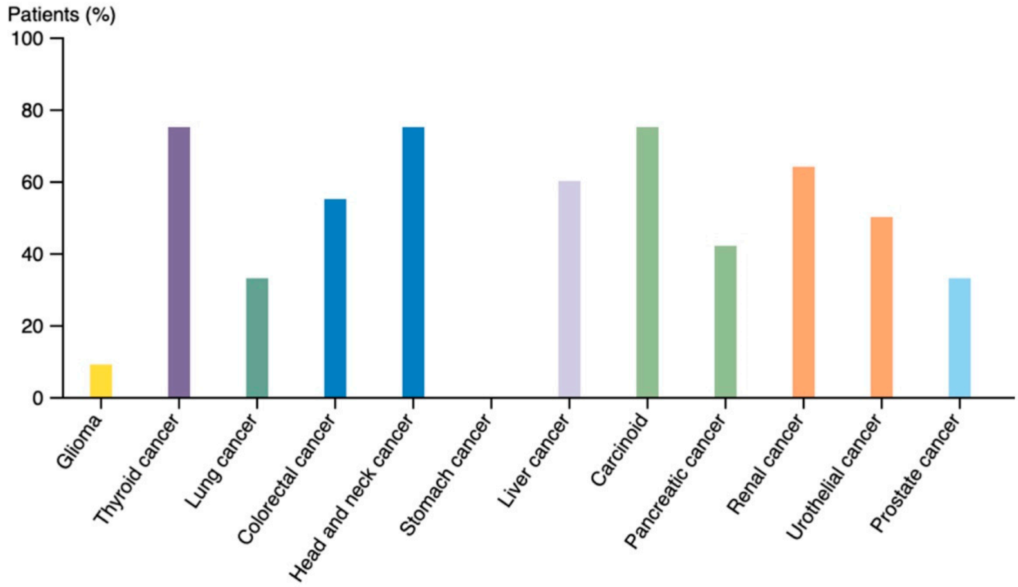

Figure S7. Analysis of fibrinogen transcripts from TCGA. Percentage of patients among each tumor grouping for above median expression of fibrinogen transcripts.

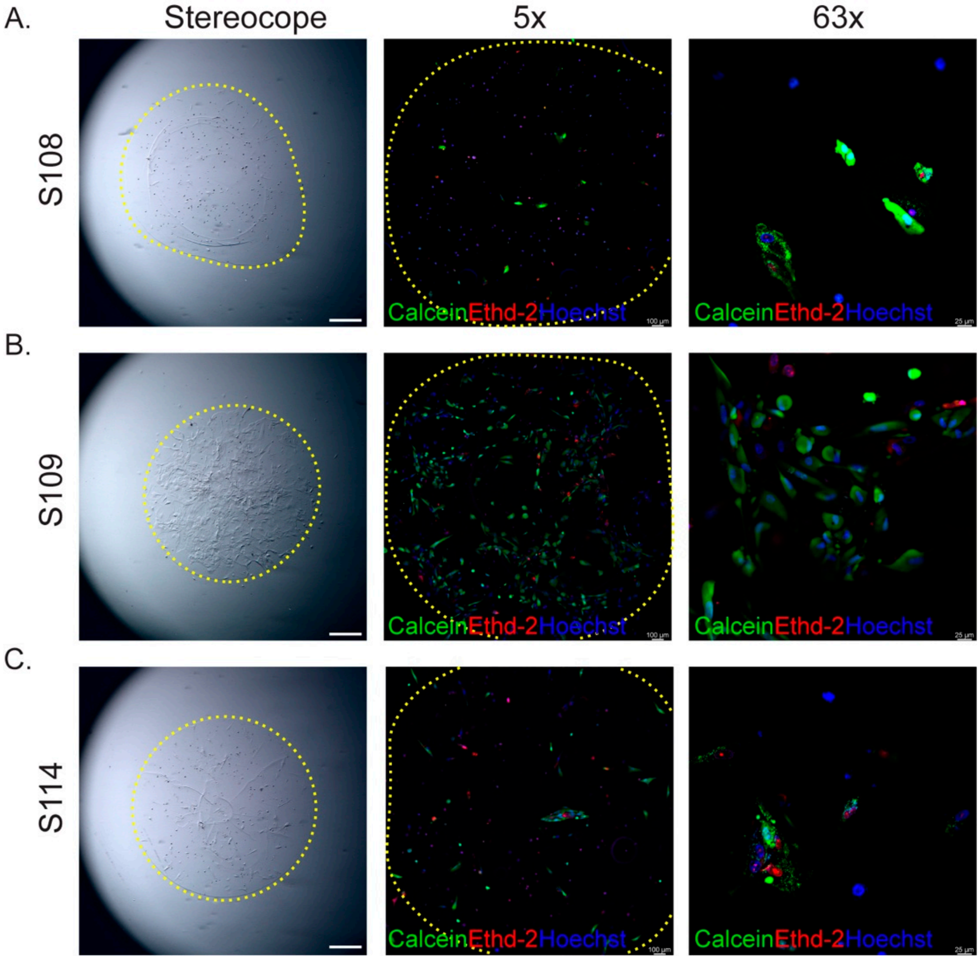

Figure S8. Primary RCC cultures create viable structures in fibrin/ccRCC ECM after 3 weeks. Yellow dotted line outlines region containing 3D domes. Green = calcein-AM, red = ethidium homodimer 2, blue = Hoechstt33342. Stereoscope and 5x image scalebar = 100 µm. 63x image scalebar = 25 µm.

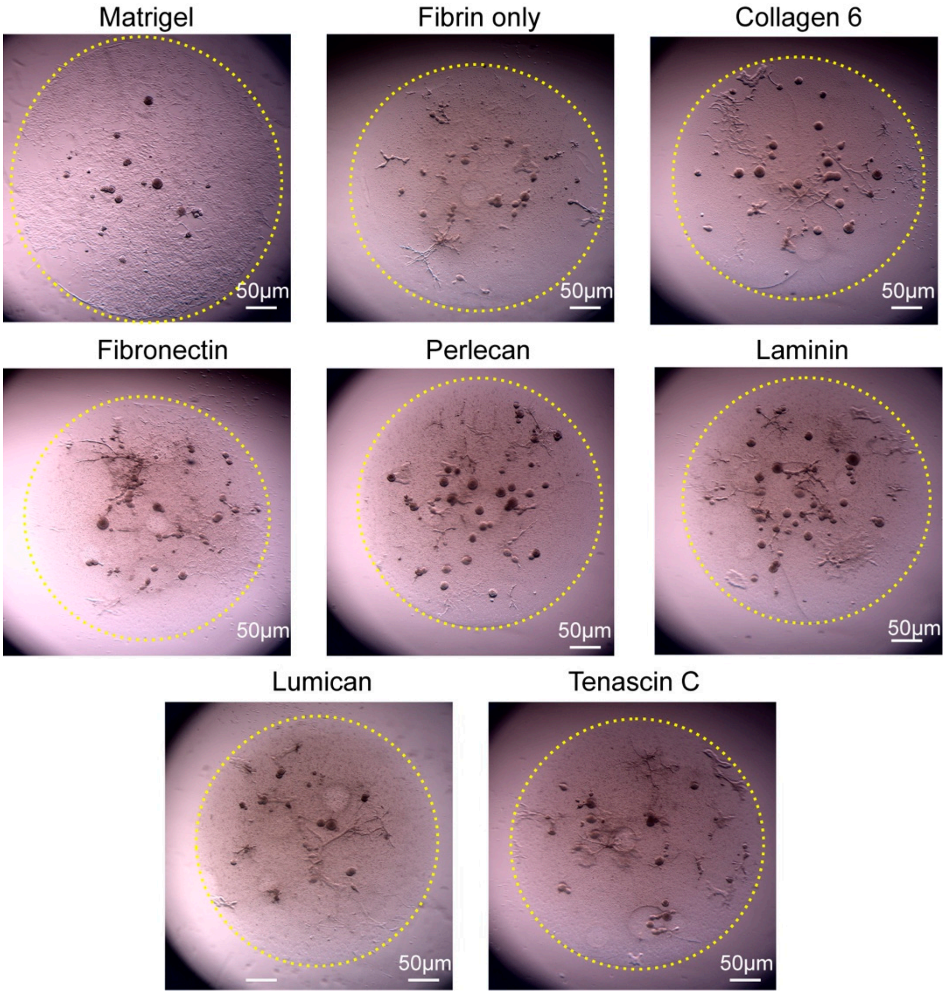

Figure S9. Incorporation of ECM into fibrin domes. Three dimensional growth of aggregates of 786-O cells in fibrin with ECM protein additives after 7 days. Yellow dotted line outlines region containing 3D dome.

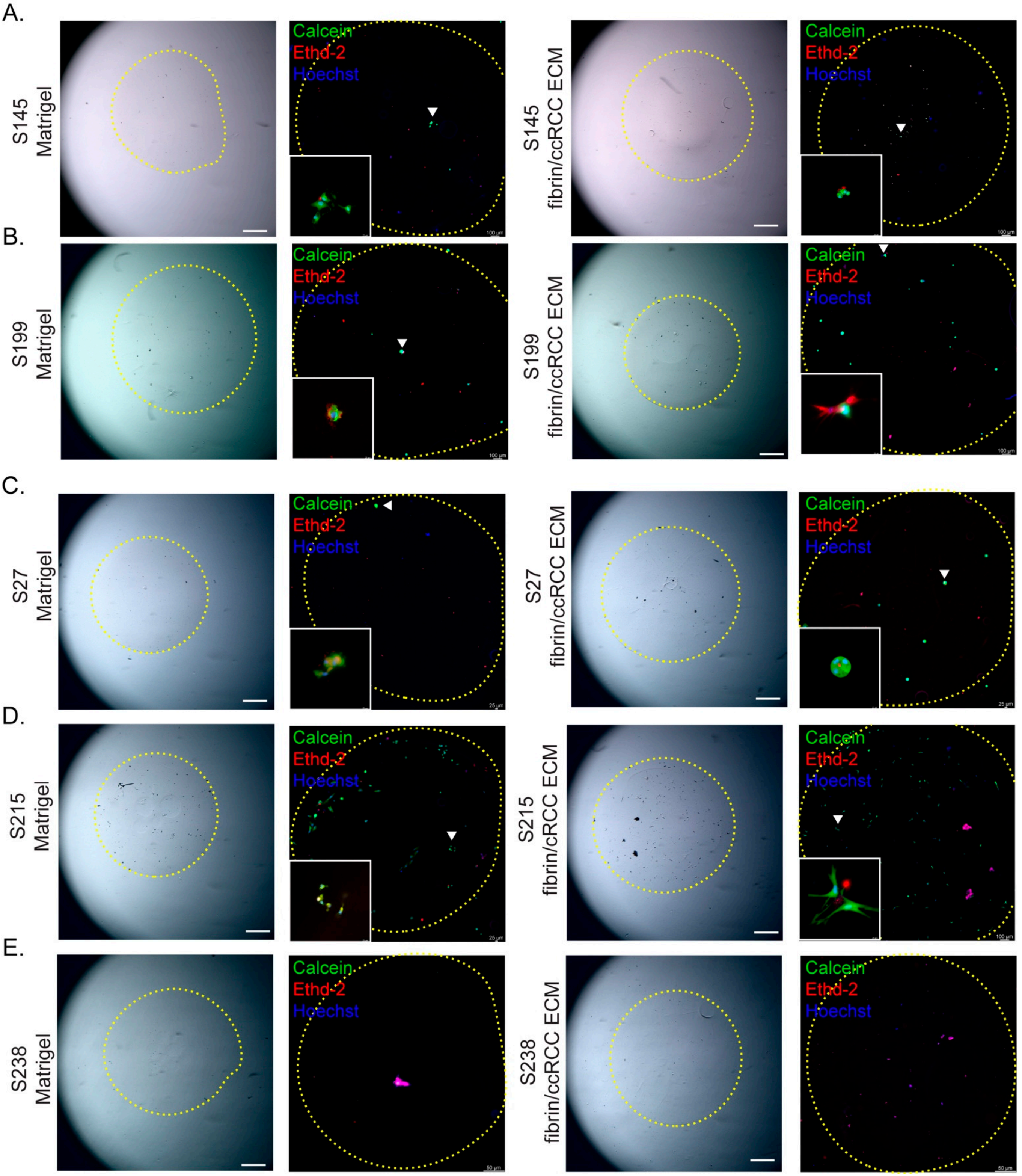

Figure S10. ccRCC tumor isolate culture in Matrigel vs fibrin/ccRCC ECM (A-E) Viability staining of primary tumor cells in Matrigel or fibrin/ccRCC ECM. Arrowhead indicates region containing inset images. Green = calcein-AM, red = ethidium homodimer 2, blue = Hoechst33342. Scale bar = 100 μm. Yellow dotted line outlines region containing 3D dome.

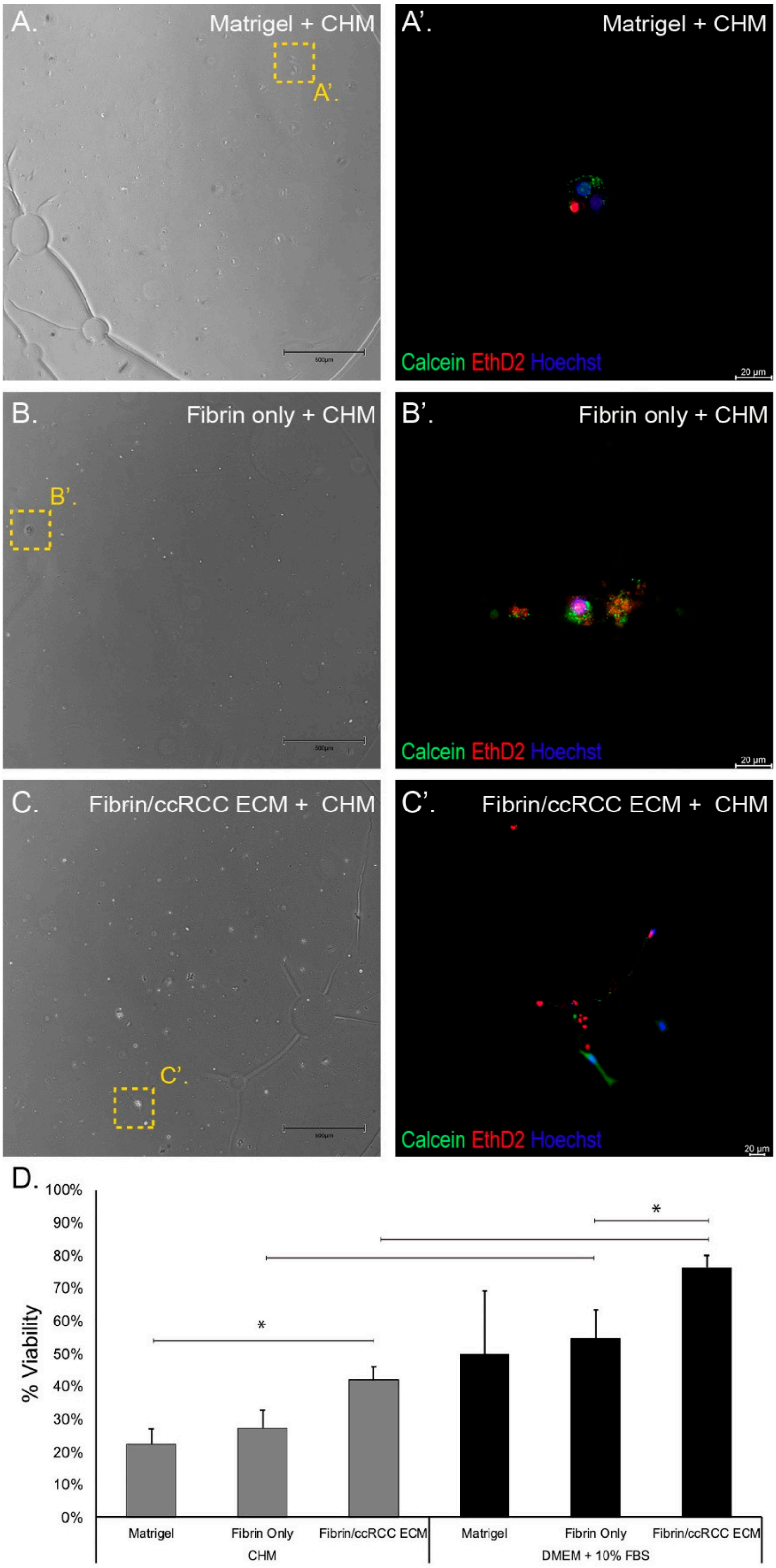

Figure S11. Media formulation influences cell viability of 3D cultures. 3D cultures from tumor sample S215 were created in either Matrigel (**A**–**A′**), Fibrinogen only (**B**–**B′**), or Fibrin/ccRCC ECM mix (**C**–**C′**) and cultured in CHM media for 14 days and analyzed for cell viability using Calcein-AM and Ethidium Homodimer-2. Yellow dotted line indicates region magnified for cell viability. Green = Calcein-AM, red = ethidium homodimer-2, blue = Hoechst 33342. (**D**) Quantification of cell viability of cultures grown in Matrigel, fibrin only, and fibrin/ccRCC ECM grown in either CHM media of DMEM + 10% FBS. * = p-value < 0.05.

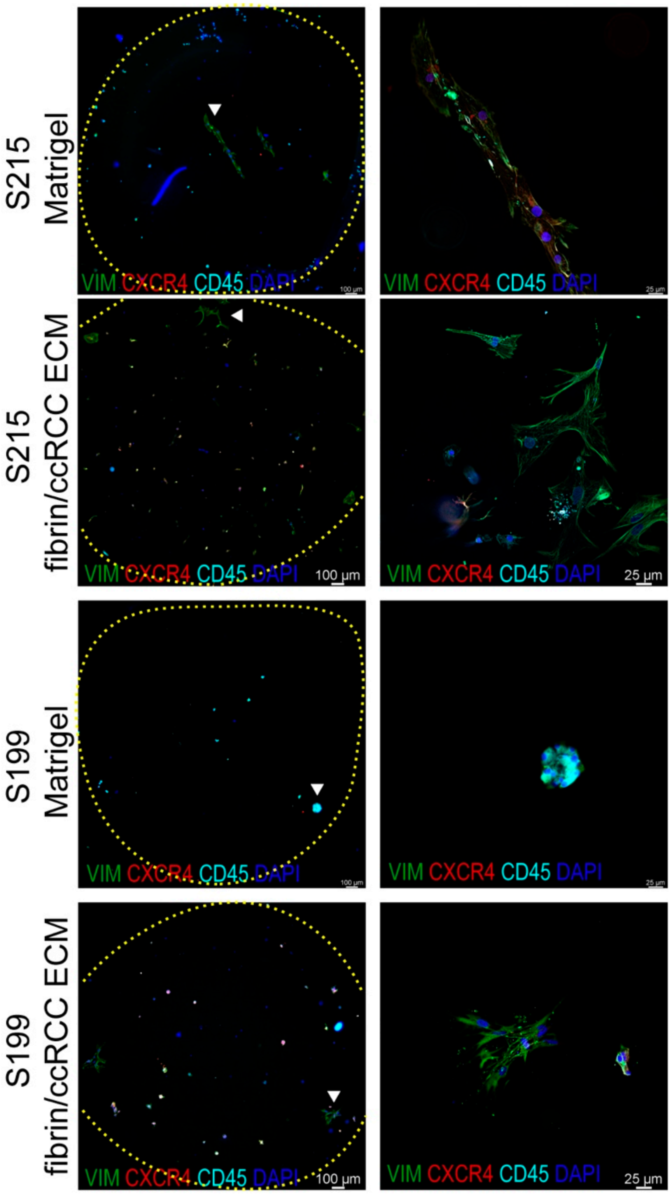

Figure S12. Further characterization of 3D cultures. Primary tumor digests grown in either Matrigel or fibrin/ccRCC ECM domes show staining for vimentin (green) in majority of cells. Subsets of vimentin-positive cells show staining for CXCR4 (red), with or without expression of CD45 (cyan). Blue = DAPI.

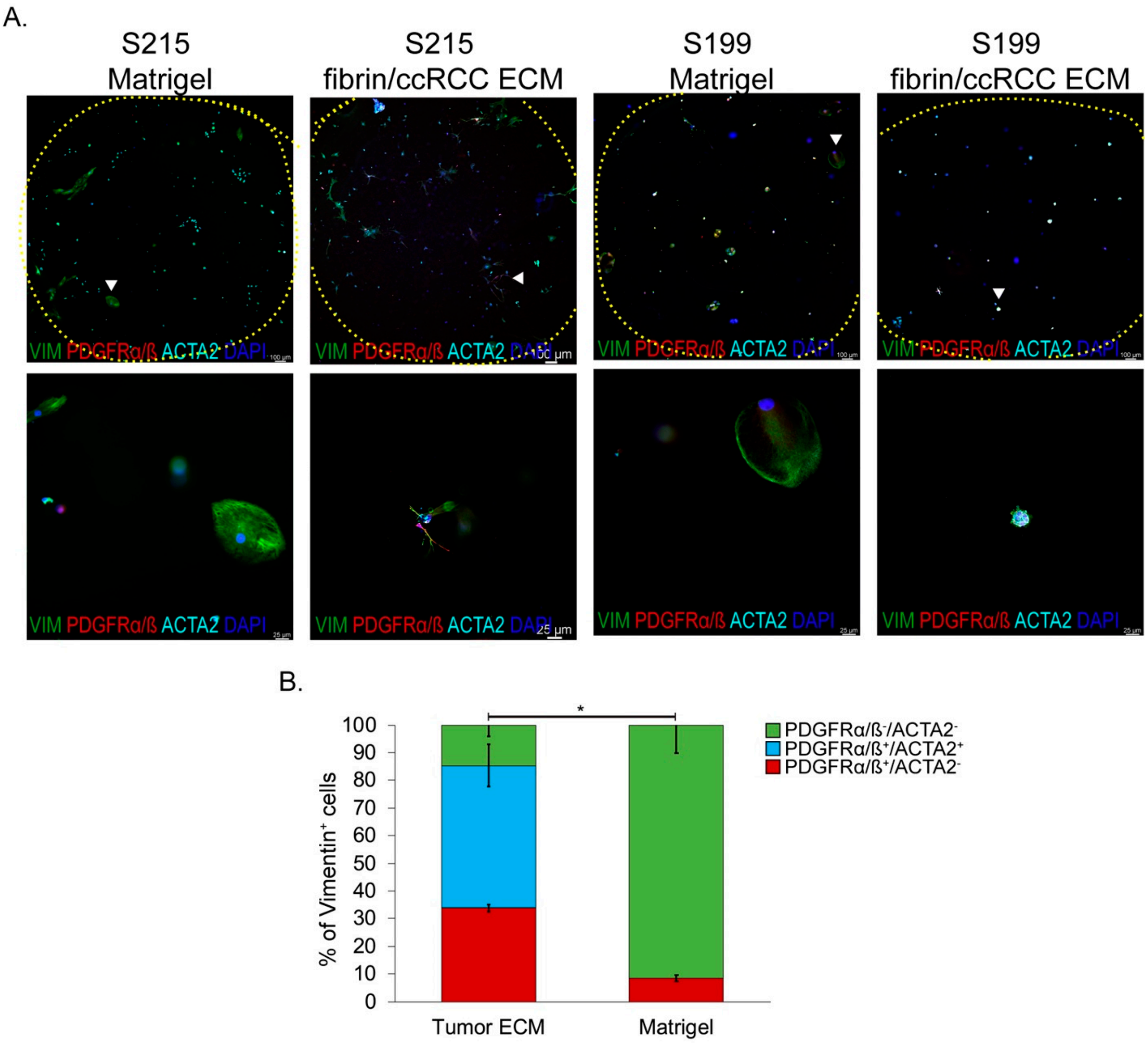

Figure S13. Immunostaining analysis of fibrin/ccRCC ECM cultures for fibroblast markers. (**A**) Staining of primary tumor digests from two different patients grown in either Matrigel or fibrin/ccRCC ECM. Arrows indicate location of magnified images below. Green = vimentin, red = PDGFRα/β, cyan = ACTA2, blue = DAPI. Scale bar top panels = 100 µm. Scale bar bottom panels = 25 µm. (**B**) Quantification of vimentin positive structures for co-staining of fibroblast marker PDGFRα/β and CAF marker ACTA2. * $p <= 0.05$ between percentage of PDGFRα/β+/ACTA2+ cells between cultures grown in fibrin/ccRCC ECM compared to Matrigel.

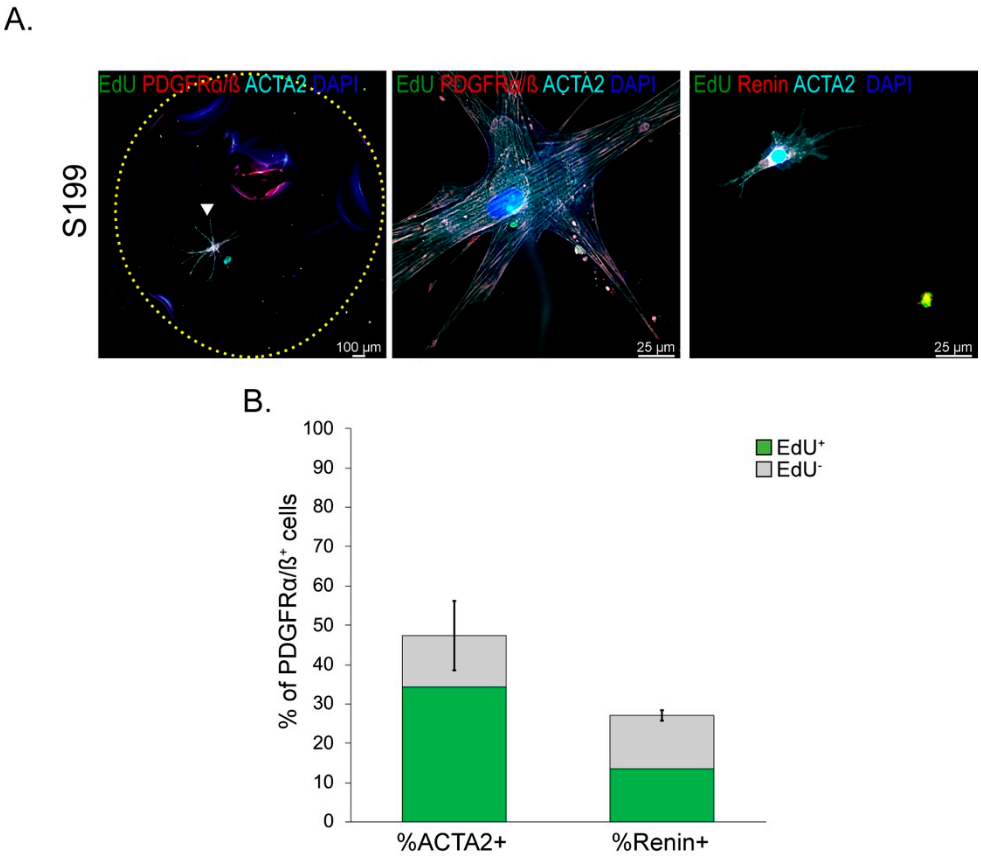

Figure S14. Additional analysis of fibroblast markers after 90 days of culture. (**A**) Immunostaining and EdU detection in sample S199 grown in fibrin/ccRCC ECM after 90 days. Yellow dotted line outlines region containing fibrin dome. White arrowhead indicates magnified region in second panel. Green = EdU, red = PDGFRα/β or renin, cyan = ACTA2, blue = DAPI. (**B**) Quantification of EdU positivity among PDGFRα/β+ cells. Percentage of cells co-positive with ACTA2 or renin additionally quantified.

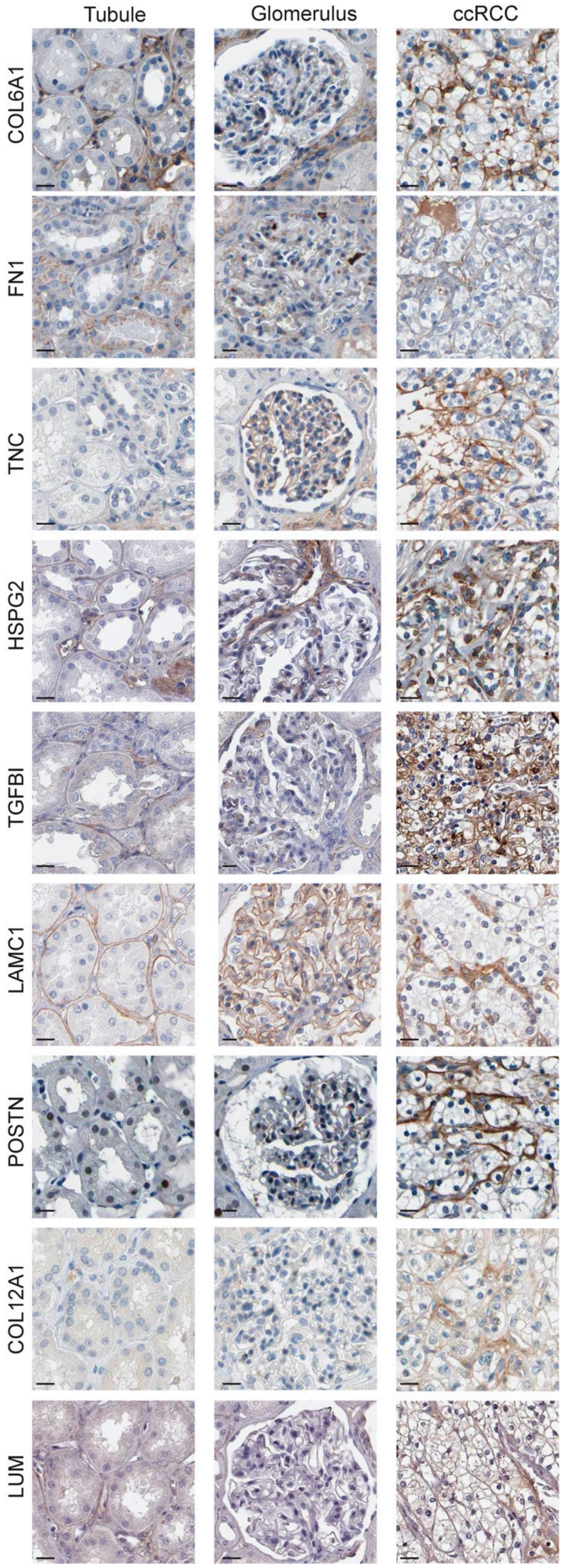

Figure S15. Protein localization in tissue. Expression patterns of ECM proteins identified in this study analyzed using IHC from ProteinAtlas. Immunostaining patterns of ECM molecules in healthy kidney tissue versus ccRCC. Healthy kidneys were investigated for staining around tubules (first column) and glomeruli (second column).
